# Minichromosome Maintenance 2 Bound with Retroviral Gp70 Is Localized to Cytoplasm and Enhances DNA-Damage-Induced Apoptosis

**DOI:** 10.1371/journal.pone.0040129

**Published:** 2012-06-29

**Authors:** Shinya Abe, Morito Kurata, Shiho Suzuki, Kouhei Yamamoto, Ken-ichi Aisaki, Jun Kanno, Masanobu Kitagawa

**Affiliations:** 1 Department of Comprehensive Pathology, Graduate School of Medical and Dental Sciences, Tokyo Medical and Dental University, Tokyo, Japan; 2 Division of Cellular and Molecular Toxicology, National Institute of Health Sciences, Tokyo, Japan; Institute for Virus Research, Laboratory of Infection and Prevention, Japan

## Abstract

The interaction of viral proteins with host-cellular proteins elicits the activation of cellular signal transduction pathways and possibly leads to viral pathogenesis as well as cellular biological events. Apoptotic signals induced by DNA-damage are remarkably up-regulated by Friend leukemia virus (FLV) exclusively in C3H hosts; however, the mechanisms underlying the apoptosis enhancement and host-specificity are unknown. Here, we show that C3H mouse-derived hematopoietic cells originally express higher levels of the minichromosome maintenance (MCM) 2 protein than BALB/c- or C57BL/6-deriverd cells, and undergo more frequent apoptosis following doxorubicin-induced DNA-damage in the presence of the FLV envelope protein gp70. Dual transfection with *gp70/Mcm2* reproduced doxorubicin-induced apoptosis even in BALB/c-derived 3T3 cells. Immunoprecipitation assays using various deletion mutants of MCM2 revealed that gp70 bound to the nuclear localization signal (NLS) 1 (amino acids 18–24) of MCM2, interfered with the function of NLS2 (amino acids 132–152), and suppressed the normal nuclear-import of MCM2. Cytoplasmic MCM2 reduced the activity of protein phosphatase 2A (PP2A) leading to the subsequent hyperphosphorylation of DNA-dependent protein kinase (DNA-PK). Phosphorylated DNA-PK exhibited elevated kinase activity to phosphorylate P53, thereby up-regulating *p53*-dependent apoptosis. An apoptosis-enhancing domain was identified in the C-terminal portion (amino acids 703–904) of MCM2. Furthermore, simultaneous treatment with FLV and doxorubicin extended the survival of SCID mice bearing 8047 leukemia cells expressing high levels of MCM2. Thus, depending on its subcellular localization, MCM2 plays different roles. It participates in DNA replication in the nucleus as shown previously, and enhances apoptosis in the cytoplasm.

## Introduction

Because ionizing irradiation (IR) and chemical agents such as doxorubicin exhibit cell-killing activity by inducing double-strand breaks (DSBs) and *p53*-dependent apoptosis, they have been considered therapeutic tools against malignant tumors [1]–[5]. To protect normal cells from injury, tumor cell-specific induction of apoptosis would be one of the most important properties of anti-tumor therapeutics [6], [7]. To regulate the *p53*-dependent apoptosis caused by DNA-damage, an understanding of upstream activators or regulators of P53 would be vital. These pathways partly involve the phosphatidylinositol 3-kinase (PI3K)-related protein kinase (PIKK) family of enzymes [8], including ataxia telangiectasia (ATM), ATM and Rad3-related (ATR), and DNA-dependent protein kinase (DNA-PK) [9]–[13].

Viral infections are known to modify cellular processes related to DNA-damage responses or DNA synthesis [14]–[16]. We have previously shown that Friend leukemia virus (FLV) infection markedly enhances the IR-induced apoptosis of hematopoietic cells in C3H mice via P53, ATM, and DNA-PK [17]. Mice infected with FLV and then treated with a low dose of total body irradiation (TBI) exhibit severe anemia. However, *p53* knockout mice, *Atm* knockout mice, and DNA-PK-deficient SCID mice with a C3H background do not exhibit this phenotype. A comparison of the apoptotic signals after FLV infection, TBI, or FLV+TBI treatment of these mice revealed that ATM is necessary for the general signal transduction of TBI-induced apoptosis [18], while DNA-PK plays a specific role in enhancing *p53*-dependent apoptosis following FLV infection [19], [20].

The enhancement of *p53*-dependent apoptosis occurs almost exclusively in the C3H strain of mice [21]. In relation to this host-specific apoptosis-enhancement, we have previously demonstrated that the FLV-derived envelope protein gp70 enhances cellular apoptotic signaling in association with host-specific overexpressed proteins, including the minichromosome maintenance (MCM) 2 protein, resulting in the activation of DNA-PK, which phosphorylates P53 [22]. MCM2 is one of a set of 6 proteins (MCM complex; MCM2-7) that play essential roles in DNA replication [23]. The MCM complex associates with the origins of DNA replication to form part of the pre-replicative complex (preRC) [24]. Activation of the MCM complex by cyclin-dependent kinases leads to the initiation of DNA synthesis and MCM proteins also act as a replicative helicase to unwind DNA at replication forks during DNA synthesis [25], [26]. The MCM complex contains a nuclear localization signal (NLS) and a nuclear export signal (NES) [27]. The NLS is split between MCM2 and MCM3 and the NES is located in MCM3 adjacent to the NLS sequence. The transport of all MCM proteins is interdependent, suggesting that nuclear import requires the formation of the hexameric complex, which would result in the assembly of a complete NLS [28], [29]. MCM proteins are expressed in cycling cells but are down-regulated and dissociated from the chromatin in quiescent cells [30]. Thus, detection of MCM proteins has emerged as a method for evaluating the proliferative state and growth fraction in dynamic cell populations. Indeed, elevated expression of several members of the MCM complex has been reported in various malignant tumors [31], [32]. Furthermore, studies with human samples have indicated the utility of MCM2 as a proliferation marker, and a high level of MCM2 expression in malignant tumors has been associated with several clinicopathological parameters, such as advanced tumor grade, advanced stage, and poor prognosis [33]–[36]. Thus, MCM2 usually acts to support cellular proliferation. However, as described above, MCM2 enhances TBI-induced apoptosis in the presence of gp70. To determine importance of such contradictory functions of the MCM2 protein in the regulation of cellular dynamics, the molecular mechanisms underlying MCM2-induced apoptosis and MCM2-gp70 interaction need to be elucidated. An understanding of the overall functions of MCM2 would enable the molecular targeting of specific functions possibly to regulate cellular proliferation/apoptosis in a cell type-specific manner and develop a novel strategy to control tumor cell growth.

**Figure 1:**
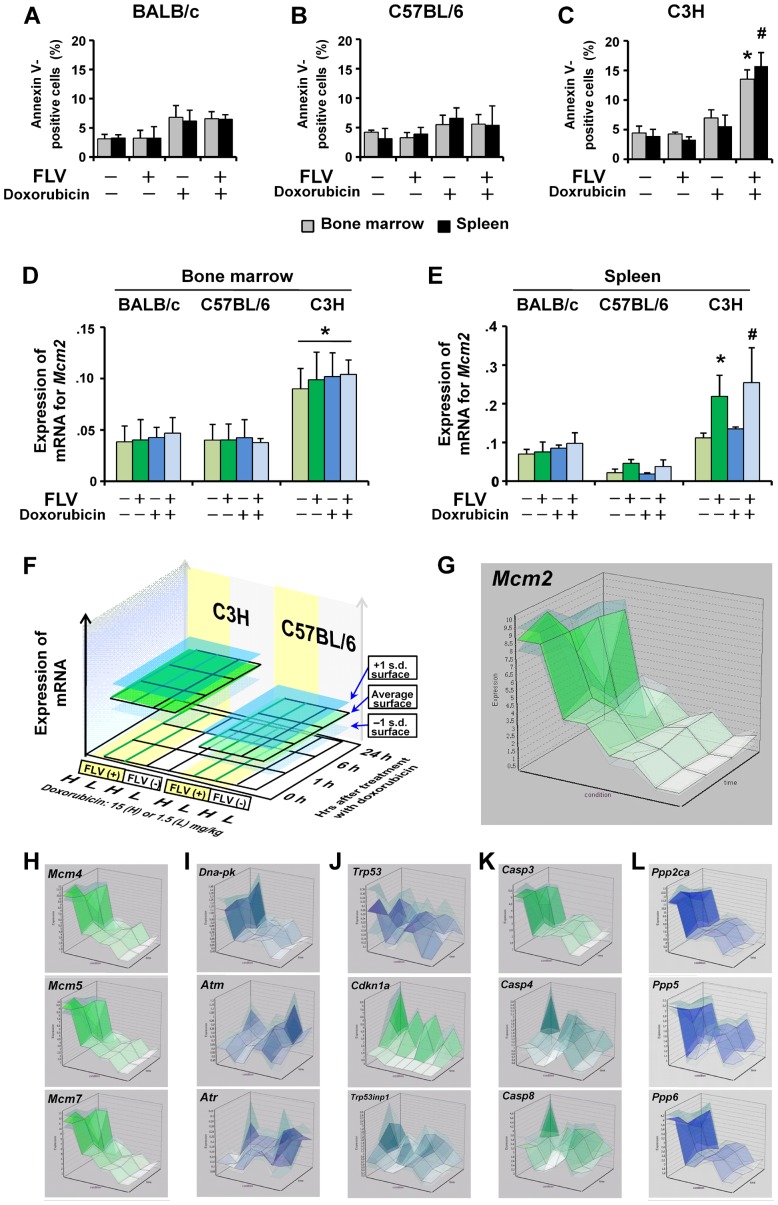
*In vivo* assessment of doxorubicin-induced apoptosis and the associated changes in mRNA expression in FLV-infected mice. Uninfected or FLV-infected BALB/c (A), C57BL/6 (B), and C3H (C) mice were intraperitoneally (i.p.) administrated with 1.5 mg/kg of doxorubicin or PBS, and the apoptotic cell ratios in the bone marrow (gray bars) and spleen cells (black bars) were determined 24 h later with annexin V-staining. Note the significant increase in the proportion of annexin V-positive cells in the bone marrow and spleen of FLV-infected C3H mice after the doxorubicin treatment compared to that in the bone marrow and spleen cells of uninfected mice “FLV (−), Doxorubicin (−)” (*p<0.01 and ^#^ *p*<0.01). Data represent the mean and 95% confidence intervals (CI) of 3 independent experiments. (D) Quantitative RT-PCR analysis of *Mcm2* mRNA expression in the bone marrow of uninfected and FLV-infected BALB/c, C57BL/6, and C3H mice. The bone marrow cells of the C3H strain exhibit higher levels of *Mcm2* in all groups compared to the corresponding groups of BALB/c and C57BL/6 mice (*p<0.01, for each group). (E) Quantitative RT-PCR analysis of *Mcm2* mRNA expression in the spleen of uninfected and FLV-infected BALB/c, C57BL/6, and C3H mice. Spleen *Mcm2* expression is higher in the “FLV (+), Doxorubicin (−)” and “FLV (+), Doxorubicin (+)” C3H mice than in the corresponding groups of BALB/c and C57BL/6 mice (*p<0.01 and ^#^ *p*<0.01, respectively). In C3H mice, FLV-infection induces higher levels of *Mcm2* expression compared to the expression in uninfected mice. Data represent the mean and 95% CI from 5 mice in each group and are representative of 2 independent experiments. The GeneChip data for *Mcm*-associated and apoptosis-associated genes were analyzed using the Percellome method. Forty-eight male C57BL/6 and C3H mice were divided into 16 groups of 3 mice each. Uninfected or FLV-infected C57BL/6 and C3H mice were administered (i.p.) with 15 mg/kg (high dose) or 1.5 mg/kg (low dose) of doxorubicin, and the spleen was sampled 0, 1, 6, and 24 h after administration. The spleen transcriptome was measured using the Affymetrix Mouse 430-2 GeneChip. (F) The Percellome data were plotted on 3-dimensional graphs for average, +1 SD, and −1 SD surfaces as demonstrated in the left schema. The scale of expression (vertical axis) is the copy number per cell. The x-axis of the 3-dimensional graph shows the experimental groups, including the C3H and C57BL/6 mice with doxorubicin treatment (high and low doses) with or without FLV-infection. The y-axis shows the time course (0, 1, 6, and 24 h) after treatment with doxorubicin and the z-axis (vertical) indicates the intensity of mRNA expression of each gene. The data of each point are connected to form a surface illustration. The expression patterns of genes are compared using the surface images. (G) The *Mcm2* expression pattern is shown in the upper right box. Of the lower columns, the first column (H) shows the data for the genes of representative *Mcm* family members, the second column (I), PI3K members, the third column (J), p53-associated genes, the fourth column (K), caspase members and fifth column (L), protein phosphatase members (PPs). *Mcm* family members, *Dna-pk*, *caspase-3* (*Casp3*), *Ppp2ac,* and *Ppp6* exhibit gene expression patterns similar to that of *Mcm2*.

## Results

### Doxorubicin-induced Apoptosis of FLV-infected Cells Correlates with High Levels of *Mcm2 in Vivo*

In previous studies, TBI caused prominent apoptosis in the bone marrow cells of FLV-infected C3H mice, but not FLV-infected BALB/c and C57BL/6 mice [17]. From a therapeutic perspective, systemic distribution of the effects of DNA-damage would be more easily achieved by chemical agents than IR. Therefore, to determine whether DNA-damaging agents enhanced apoptosis to similar extents in FLV-infected mice of different strains, uninfected or FLV-infected BALB/c, C57BL/6, and C3H mice were intraperitoneally administered with a low dose of doxorubicin or PBS, and the apoptotic cell ratio was measured in the bone marrow and spleen. In FLV-infected BALB/c and C57BL/6 mice, the apoptotic cell ratios after treatment with doxorubicin were similar to the ratios in uninfected mice (Figure 1A, B). On the other hand, FLV-infected doxorubicin-treated C3H mice exhibited significantly higher ratios with uninfected mice (Figure 1C). Thus, we could generalize as to the effects of DNA-damage by IR and chemical agents on the enhancement of apoptosis by FLV-infection in hematopoietic organs.

Next, we examined the expression of *Mcm2* mRNA in the bone marrow and spleen of uninfected and FLV-infected BALB/c, C57BL/6, and C3H mice. *Mcm2* levels were significantly higher in the bone marrow cells of C3H mice than in BALB/c and C57BL/6 mice (Figure 1D). Spleen *Mcm2* levels were also higher in C3H mice than in BALB/c and C57BL/6 mice. Furthermore, in C3H mice, the spleen *Mcm2* levels were elevated by FLV-infection (Figure 1E). Similar trends were observed across all the inbred strains tested. These results suggest that doxorubicin treatment induces significant apoptosis in FLV-infected C3H mice in association with higher levels of *Mcm2*. Moreover, we performed a comparative GeneChip analysis using RNA isolates from mouse spleen and identified several genes that exhibited various expression patterns in the different mouse strains (Figure 1F-L). *Mcm2* expression was higher in C3H mice than in C57BL/6 mice, and *Mcm2* expression was elevated by FLV-infection (Figure 1G). Genes that exhibited expression patterns similar to that of *Mcm2* are listed in Table S1.

### Dual Transfection with *Mcm2/gp70* Enhances DNA-damage-induced Apoptosis in BALB/c-derived 3T3 Cells

**Figure 2:**
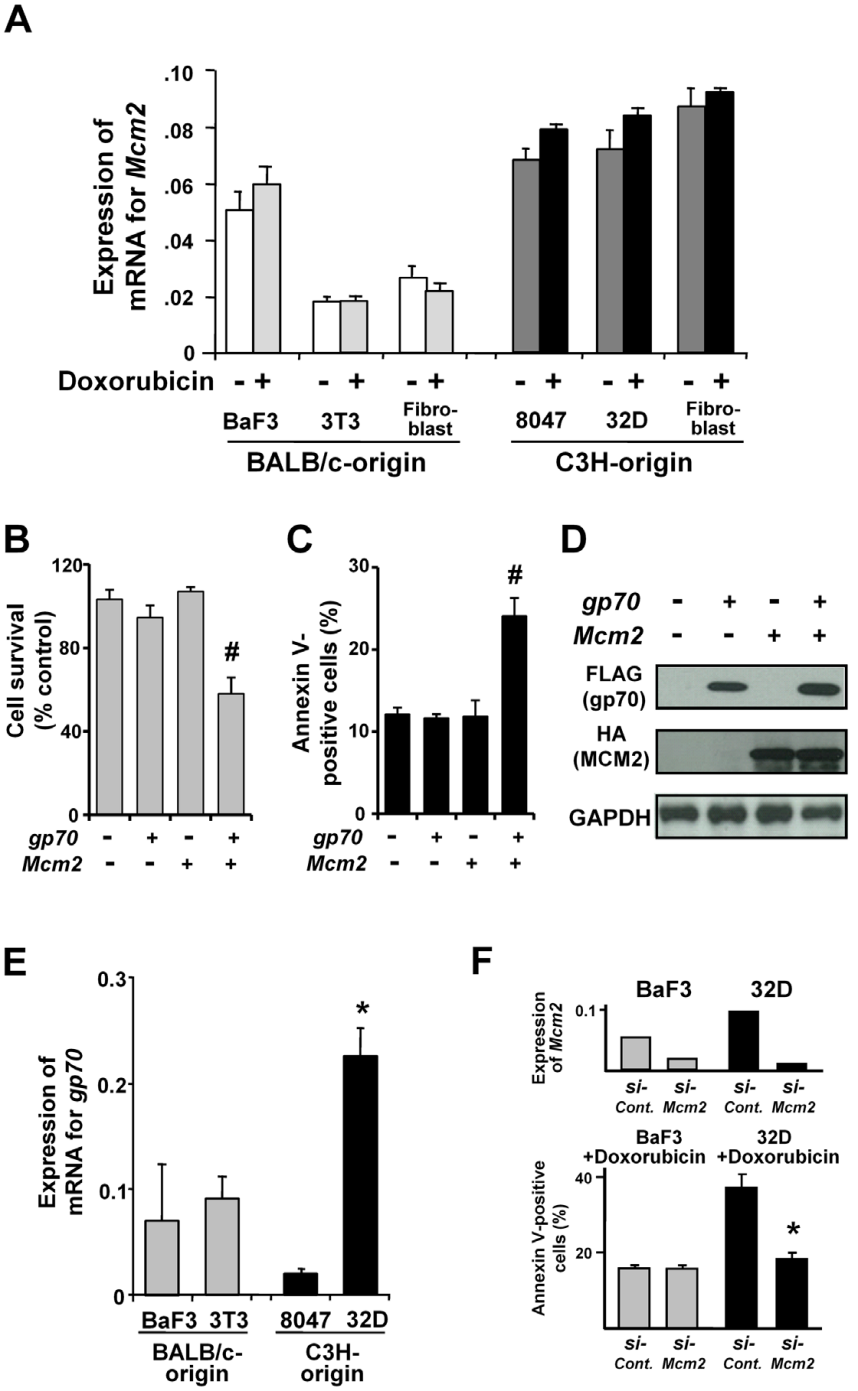
Dual transfection of *gp70* and *Mcm2* enhances DNA-damage-induced apoptosis in 3T3 cells. (**A**) Quantitative RT-PCR analysis of *Mcm2* mRNA expression in untreated and doxorubicin-treated BALB/c-derived BaF3 and 3T3 cells, and primary cultured fibroblasts, and C3H-derived 8047 and 32D cells, and primary cultured fibroblasts. Data represent the mean and 95% CI of 3 independent experiments. (**B**) Cell survival (% of control) measured with the MTT assay in *gp70* and/or *Mcm2*-transfected 3T3 cells after treatment with doxorubicin for 24 h. Cell survival is significantly different between control cells “*gp70* (−), *Mcm2* (−)” and *gp70/Mcm2*-transfected cells “*gp70* (+), *Mcm2* (+)” (#*p*<0.01). Data represent the mean and 95% CI of 3 independent experiments. (**C**) Apoptotic cell ratios in *gp70* and/or *Mcm2*-transfected 3T3 cells were determined with annexin V-staining after treatment with 1 µM doxorubicin for 24 h. The ratios in the control cells “*gp70* (−), *Mcm2* (−)” and *gp70/Mcm2*-transfected cells “*gp70* (+), *Mcm2* (+)” are significantly different (#*p*<0.01). Data represent the mean and 95% CI of 3 independent experiments. (**D**) Western blot analysis of *gp70* and/or *Mcm2-FL*-transfected 3T3 cells after treatment with 1 µM of doxorubicin for 24 h. Gp70 and MCM2 protein levels are similar in all groups. (**E**) Expression of endogenous *gp70* mRNA in BaF3, 3T3, 8047, and 32D cells. G*p70* mRNA expression (ng) was normalized to that of *GAPDH*. Note the significantly higher expression of *gp70* mRNA in 32D cells compared to that in the other cells (*p<0.01). Data show the mean and 95% CI of three independent experiments. (**F**) *Mcm2* knockdown in BaF3 and 32D cells using siRNA. Quantitative RT-PCR (upper) was performed to confirm *si-Mcm2*-induced reduction of *Mcm2* mRNA expression. Apoptotic cell ratios were determined with annexin V-staining after treatment with doxorubicin for 24 h (bottom). Note the significant decrease in the apoptotic cell ratio of 32D cells treated with *si-Mcm2,* compared to that of cells treated with *si-Control* (*p<0.01). Data show the mean and 95% CI of 3 independent experiments.

To investigate whether apoptosis enhancement was related to the high levels of *Mcm2* in FLV-infected cells, we analyzed doxorubicin-induced apoptosis sensitivity in *Mcm2* and/or *gp70*-transfected 3T3 cells. First, the expression of *Mcm2* was analyzed in each mouse cell line. BALB/c-derived 3T3 cells and primary cultured BALB/c-fibroblasts expressed low levels of *Mcm2* compared to C3H-derived 8047 cells, 32D cells and primary cultured C3H-fibroblasts (Figure 2A).

Next, the viability and apoptotic cell ratios of 3T3 cells were evaluated after doxorubicin treatment. *Gp70* plus *Mcm2*-transfected 3T3 cells exhibited a significant decrease in viability and an increase in apoptotic cell ratio compared to control cells, whereas cells transfected with *gp70* or *Mcm2* exhibited no significant change in viability and apoptotic cell ratio (Figure 2B, C). Gp70 and/or MCM2 protein levels following *gp70*- and/or *Mcm2*-transfection were similar in all the experimental groups (Figure 2D). Next, we knocked down the expression of *Mcm2* in BaF3 and 32D cells using siRNA. The 32D cell line, with a high level of endogenous gp70 expression, was established from FLV-infected C3H mouse bone marrow [37] (Figure 2E). *Mcm2* knockdown significantly reduced *Mcm2* mRNA expression and apoptotic cell ratio of 32D cells treated with doxorubicin in contrast to the non-remarkable change in the apoptotic cell ratio of BaF3 cells (Figure 2F). These results suggest that the host-specific enhancement of DNA-damage-induced apoptosis is associated with the higher level of *Mcm2* expression in C3H-derived cells.

### Gp70 Directly Binds to the N-terminal Portion of MCM2

**Figure 3:**
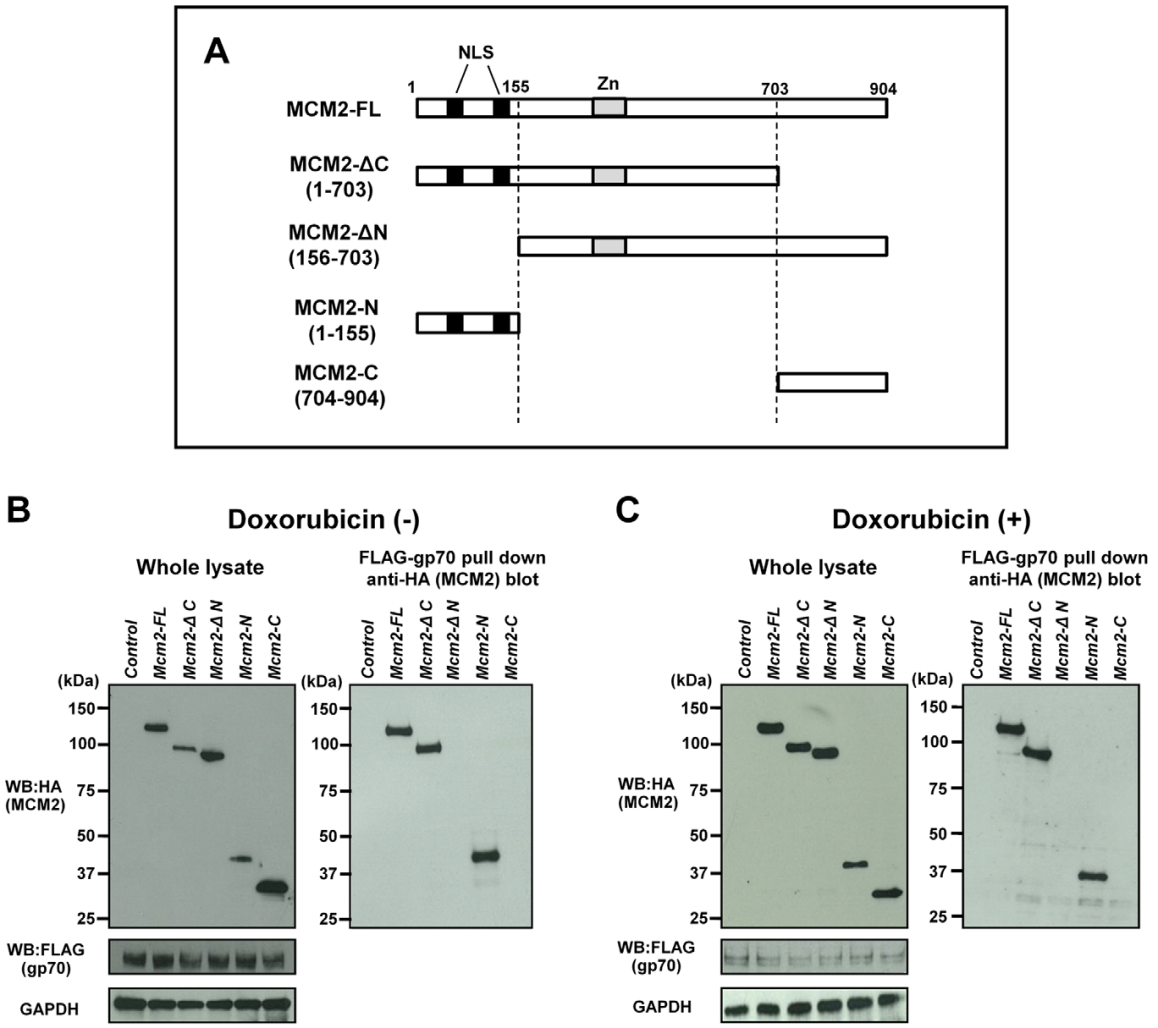
Direct interaction of MCM2 with gp70. (**A**) Schematic diagram of full-length MCM2 (MCM2-FL) and MCM2 deletion mutants, MCM2-ΔC (aa 1–703), MCM2-ΔN (aa 156–703), MCM2-N (aa 1–155) and MCM2-C (aa 704–904). The NLS domains are shown in black, and the Zn-finger domains are gray. 3T3 cells were transfected with *HA*-tagged *Mcm2* mutants along with *FLAG*-tagged *gp70*, and either left untreated (**B**) or treated with 1 µM doxorubicin for 24 h (**C**). The expression of the MCM2 mutants (**B**, **C**, left upper) and FLAG-gp70 (**B**, **C**, left middle) was confirmed in 3T3 cells. Cell lysates were subjected to a pull-down assay to detect the binding of MCM2-FL or MCM2 mutants to FLAG-gp70 (**B**, **C**, right panel).

To examine the molecular interactions between MCM2 and gp70, immunoprecipitation experiments were performed. We generated plasmids encoding HA-tagged full-length MCM2 (MCM2-FL) and various deletion mutants: MCM2-ΔC, MCM2-ΔN, MCM2-N and MCM2-C (Figure 3A). Each of these plasmids was transfected into 3T3 cells along with *FLAG*-tagged *gp70*. Irrespective of doxorubicin treatment, gp70 interacted with MCM2-FL, MCM2-ΔC, and MCM2-N, but not with MCM2-ΔN or MCM2-C (Figure 3B, C). These results indicate that gp70 associates with the N-terminal portion of MCM2. Gp70 binding inhibited the formation of the MCM complex (Figure S1). As shown in Figure 3B and 3C, the size of MCM2-N was larger than the expected size. Generally, phosphorylated proteins are sometimes larger than their unphosphorylated counterparts [38], [39]. Indeed, the N-terminal portion of MCM2 possesses many phosphorylation sites [40]. Therefore, the apparent molecular weight of MCM2-N may be higher than expected. Further, MCM2-C does not have as many phosphorylation sites [40]. As a result, MCM2-N may appear larger than MCM2-C.

We also generated plasmids encoding a FLAG-tagged gp70 deletion mutant (Figure S2A) and performed a similar pull-down assay after co-transfection with HA-tagged *Mcm2*-FL. MCM2 bound to the middle portion of gp70 (Figure S2B, C) and enhanced apoptosis in response to doxorubicin (Figure S2D, E).

### The C-terminal Portion of MCM2 is Essential for the Enhancement of Doxorubicin-induced Apoptosis

**Figure 4:**
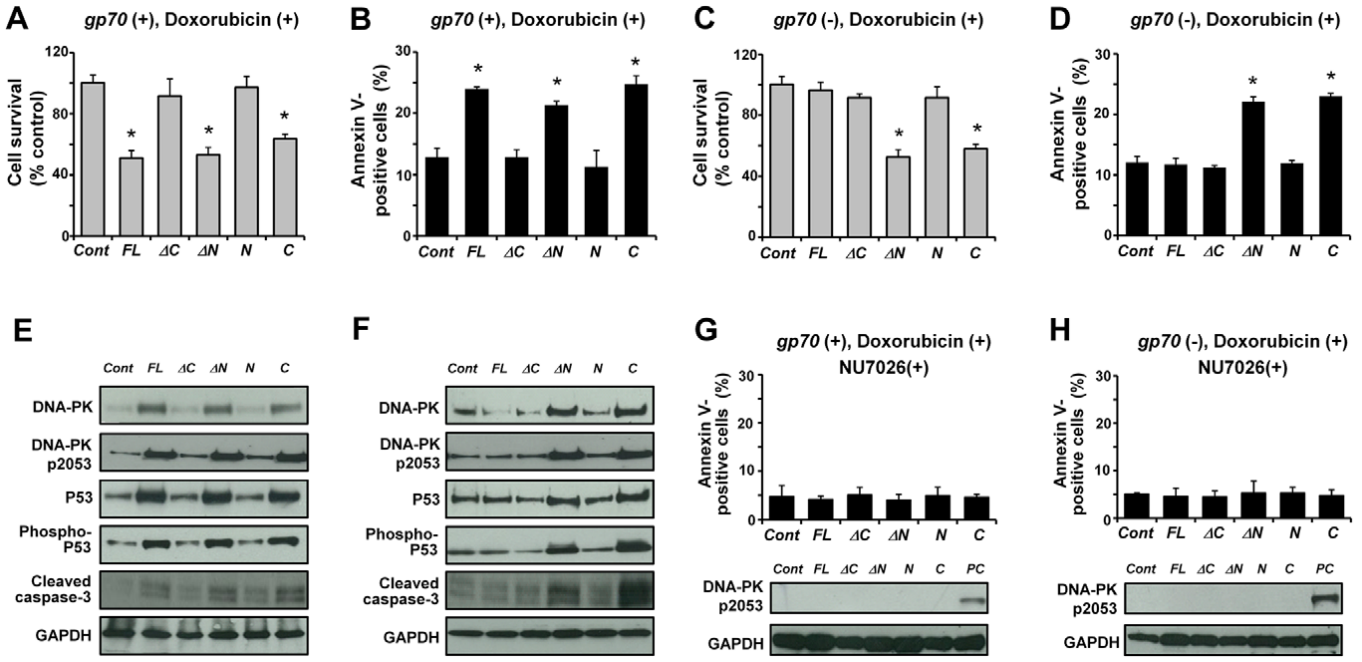
The C-terminal portion of MCM2 is important for apoptosis enhancement. 3T3 cells were co-taransfected with *gp70* and *Mcm2-FL* or the mutants (**A**, **B**) or transfected with *Mcm2-FL* or the mutants (**C**, **D**) and treated with 1 µM doxorubicin for 24 h. Cell survival (**A**, **C**) and apoptotic cell ratios (**B**, **D**) were determined using the MTT assay and annexin V-staining, respectively. Asterisks (*) indicate *p*<0.01 for control vs. mutant-transfected cells. In all panels, data represent the mean and 95% CI of 3 independent experiments. Western blot analysis of *gp70*/*Mcm2-FL-* and *gp70*/mutant-transfected 3T3 cells (**E**) and *Mcm2-FL-* and mutant-transfected 3T3 cells (**F**) after treatment with 1 µM doxorubicin for 24 h. The levels of DNA-PK, phospho-DNA-PK (pS2053), P53, phospho-P53, and cleaved caspase-3 are elevated in the groups with elevated apoptotic ratios. (**G**) 3T3 cells co-transfected with *gp70*/*Mcm2-FL* or *gp70*/mutants and (**H**) 3T3 cells transfected with *Mcm2-FL* or the mutants were pre-incubated with 10 µM NU7026, a DNA-PK-inhibitor, for 2 h and treated with 1 µM doxorubicin for 24 h. DNA-PK-pS2053 levels are substantially reduced in cells treated with the DNA-PK-inhibitor (**G** and **H**, bottom) compared to the levels in the absence of NU7026 (**E** and **F**, respectively). Whole cell lysates from *gp70-* and *Mcm2-FL*-transfected 3T3 cells after doxorubicin treatment are shown as a positive control (PC, **G** and **H**, bottom). Apoptotic cell ratios were determined with annexin V-staining (**G** and **H,** upper graph). In both panels, data represent the mean and 95% CI of three independent experiments.

Next, to identify the functional domain of MCM2 essential for apoptosis enhancement following DNA-damage, a functional analysis was performed using MCM2 deletion mutants. First, *Mcm2-FL* or the deletion mutant were introduced into 3T3 cells with or without *gp70*. After the transfection, 3T3 cells were treated with doxorubicin, and cell viability and apoptotic cell ratio were measured. 3T3 cells, transfected with *gp70* and the *Mcm2-FL* exhibited a significant decrease in viability and an increase in apoptotic cell ratio compared to cells transfected with the negative control (Figure 4A, B). Surprisingly, cells transfected with *gp70* and *Mcm2-ΔN*- or *Mcm2-C*, which did not interact with gp70, also exhibited a significant decrease in viability and an increase in apoptotic cell ratio relative to the negative control (Figure 4A, B). Among the cells singly transfected with *Mcm2-FL* or the mutants, *Mcm2-FL*-, *Mcm2-ΔC*-, and *Mcm2-N*-transfected cells exhibited no remarkable change in viability and apoptotic cell ratio compared to the negative control (Figure 4C, D). By contrast, *Mcm2-ΔN* and *Mcm2-C*-transfected cells exhibited a significant decrease in viability and an increase in apoptotic cell ratio (Figure 4C, D).

Previous studies have shown that MCM2 is essential for DNA replication [23], [25], and its expression is up-regulated in proliferating cells [41]. *Mcm2*-transfected 3T3 cells exhibited no significant change in cell count during the early stage (Figure S3A, B). However, at a later-stage (96 h), the cell count was significantly higher in *Mcm2*-transfected 3T3 cells than in the control (Figure S3C, D).

We next examined the protein levels of DNA-PK, phospho-DNA-PK (pS2053), P53, phospho-P53, and cleaved caspase-3 in *Mcm2-FL*- or Mcm2 deletion mutant-transfected 3T3 cells after doxorubicin treatment. Among the cells transfected with *gp70* plus *Mcm2-FL*- or *gp70* plus mutant-transfected cells, *Mcm2-FL*-, *Mcm2-ΔN*-, and *Mcm2-C*-transfected cells expressed higher endogenous levels of DNA-PK, phospho-DNA-PK, P53, phospho-P53, and cleaved caspase-3 than the negative control (Figure 4E). By contrast, the levels of these proteins in *Mcm2-ΔC*- and *Mcm2-N*-transfected cells did not change (Figure 4E). Among the cells singly transfected with *Mcm2-FL* or a mutant, *Mcm2-ΔN*-, and *Mcm2-C*-transfected cells exhibited higher levels of DNA-PK, phospho-DNA-PK, P53, phospho-P53, and cleaved caspase-3 after doxorubicin treatment (Figure 4F). These results indicate that not only the binding of MCM2 with gp70 but also deletion of the N-terminal portion enhances DNA-damage-induced apoptosis via the activation of P53 by DNA-PK. Furthermore, MCM2 lacking the C-terminal portion did not induce apoptosis even with *gp70* co-expression indicating that the C-terminal portion of MCM2 was essential for the enhancement of DNA-damage-induced apoptosis.

DNA-PK is robustly activated by auto-phosphorylation at Ser 2056 (S2053 in mouse) in apoptotic cells [42], while phosphorylation at Thr 2609 is associated with non-homologous end joining [43]. Therefore, to examine whether DNA-PK was exclusively required for the enhancement of apoptosis, we inhibited DNA-PK activity using NU7026 in the presence (Figure 4G) or absence of *gp70* (Figure 4H). Inhibition of DNA-PK activity by NU7026 substantially reduced the level of phospho-DNA-PK (pS2053) and completely abolished apoptosis enhancement in cells expressing the *Mcm2 mutants* (Figure 4G, H). These results and knockdown experiments (Figure S4) indicate that DNA-PK activation is necessary for the enhancement of doxorubicin-induced apoptosis.

### The Gp70-MCM2 Complex Binds to PP2A and Causes Hyperphosphorylation of DNA-PK

**Figure 5:**
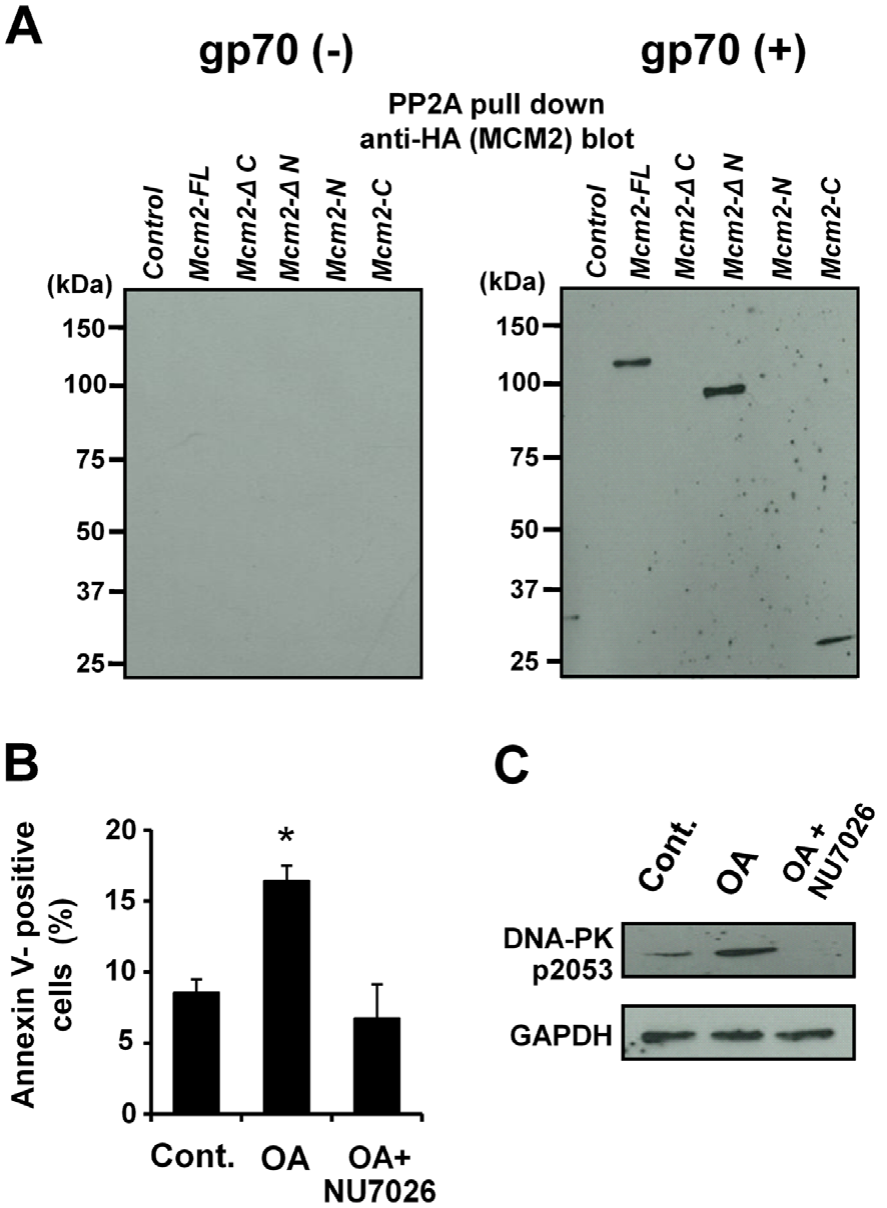
MCM2 (FL and mutants) interacts with PP2A. (**A**) The *Mcm2-FL*- or mutant-transfected 3T3 cells (left) and *gp70*/*Mcm2-FL-* or *gp70*/mutants-transfected 3T3 cells (right) were treated with 1 µM doxorubicin for 24 h. Cell lysates were subjected to a pull-down assay to detect the binding of MCM2-FL or the mutants to PP2A. (**B**) 3T3 cells were pre-incubated with 10 nM okadaic acid (OA) and 10 µM NU7026 for 2 h, and treated with 1 µM doxorubicin for 24 h. The apoptotic cell ratio was determined with annexin V-staining. Asterisk (*) indicates *p*<0.01 for control vs. mutant-transfected cells. Data represent the mean and 95% CI of 3 independent experiments. (**C**) Western blot analysis of 3T3 cells to detect phospho-DNA-PK. Note the significantly increased levels of DNA-PK-p2053 in OA-treated 3T3 cells, and the complete abrogation by NU7026.

To determine the mechanism by which the gp70-MCM2 complex activated DNA-PK to enhance apoptosis, we sought to identify the upstream regulatory factors of DNA-PK. We focused on protein phosphatase 2A (PP2A), because this molecule has been shown to dephosphorylate DNA-PK and control its function [44]–[46]. 3T3 cells were transfected with *Mcm2-FL* or *Mcm2* deletion mutants with or without *gp70* and treated with doxorubicin. In the absence of *gp70*, PP2A did not interact with MCM2-FL or the mutants (Figure 5A, left). In *gp70*-transfected cells, PP2A co-precipitated with MCM2-FL, MCM2-ΔN, and MCM2-C, but not with MCM2-ΔC or MCM2-N (Figure 5A, right). Thus, PP2A interact with the C-terminal portion of MCM2 in *gp70*-transfected 3T3 cells.

To determine whether the enhanced apoptosis was caused by the inactivation of PP2A, the PP2A-specific inhibitor okadaic acid (OA) was added to 3T3 cells that were treated with doxorubicin. As expected, the OA-treated 3T3 cells exhibited a significant increase in apoptotic cell ratio compared to the control (Figure 5B). Furthermore, NU7026 treatment abrogated the doxorubicin-induced apoptosis enhancement in OA-treated 3T3 cells (Figure 5B). The expression of phospho-DNA-PK (pS2053) was upregulated in OA-treated 3T3 cells after doxorubicin treatment (Figure 5C). These results suggest that the gp70-MCM2 complex binds to and inhibits PP2A. Consequently, DNA-PK is hyperphosphorylated and doxorubicin-induced apoptosis is enhanced via the P53/cleaved caspase-3 pathway.

### The gp70-MCM2 Complex is Localized in the Cytoplasm

**Figure 6:**
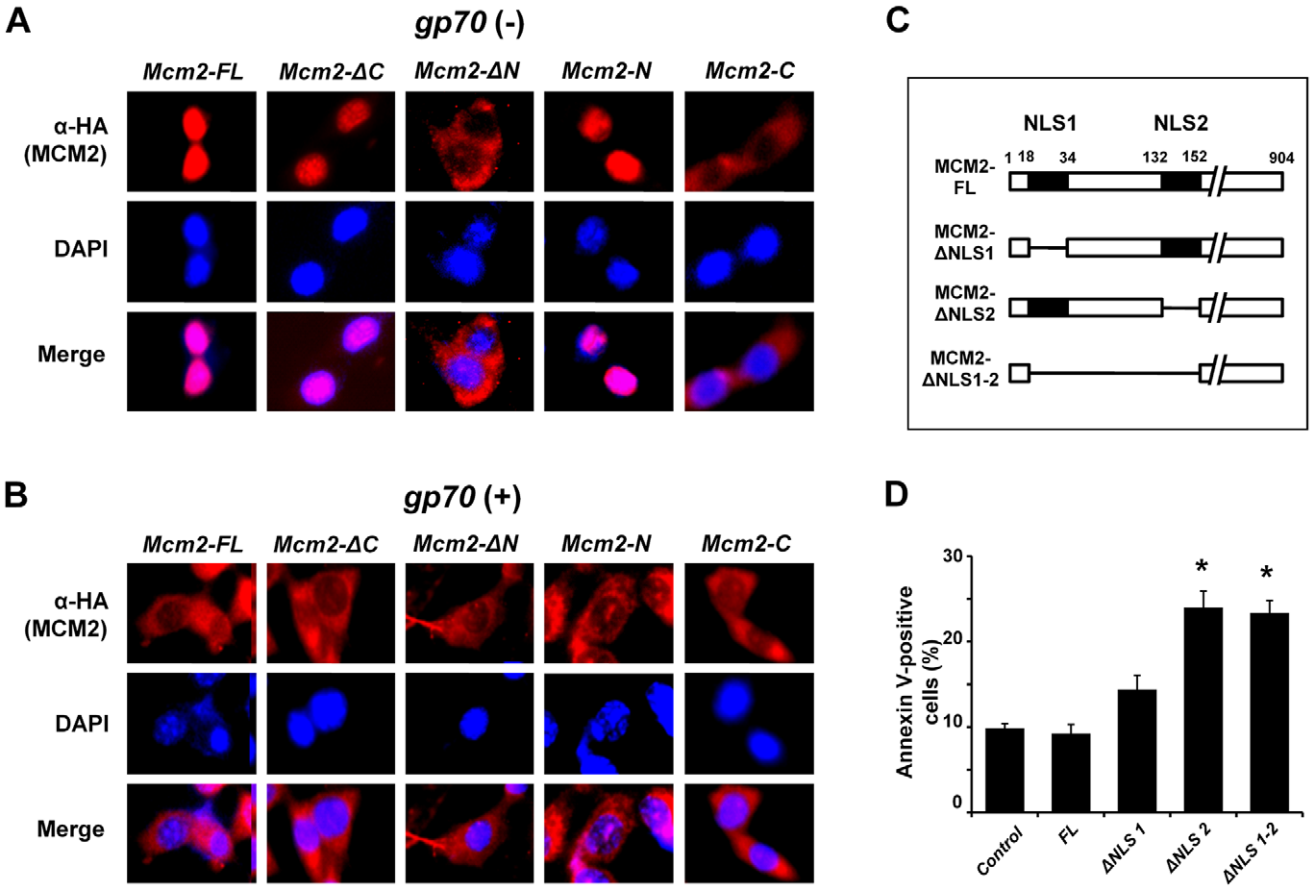
Subcellular localization of MCM2 and the role of the NLS domains in enhancing doxorubicin-induced apoptosis. *HA-Mcm2-FL* and *HA-*mutant-transfected 3T3 cells (**A**), and *FLAG-gp70*/*HA-Mcm2-FL* and *FLAG-gp70*/*HA*-mutant-transfected 3T3 cells (**B**) were treated with 1 µM doxorubicin for 24 h. HA-positive cells containing the MCM2-derived proteins are shown in red (TRITC), and DAPI-stained nuclei are shown in blue. Images were acquired using a BZ-9000 microscope (KEYENCE) with a 400× objective. (**C**) Schematic diagram of the NLS deletion mutants MCM2-ΔNLS1, MCM2-ΔNLS2, and MCM2-ΔNLS1-2. (**D**) *Mcm2-NLS* deletion mutant-transfected 3T3 cells were treated with 1 µM doxorubicin for 24 h, and apoptotic cell ratios were determined with annexin V-staining. Data represent the mean and SD of 3 experiments. The asterisks (*) indicate significant differences between the control and *Mcm2-ΔNLS2*- or *Mcm2-ΔNLS 1-2*-transfected cells (*p<0.01). Data represent the mean and 95% CI of 3 independent experiments.

The MCM2 protein contains an NLS in the N-terminal portion. Thus, MCM2 localizes to the nucleus when expressed in HeLa cells [47]. To investigate the cellular localization of MCM2, immunofluorescence was performed on 3T3 cells transfected with *Mcm2-FL* or mutated *Mcm2*, with or without *gp70* and treated with doxorubicin. In 3T3 cells singly transfected with *Mcm2-FL* or the mutants, MCM2-FL as well as MCM2-ΔC and MCM2-N were localized in the nucleus (Figure 6A). By contrast, MCM2-ΔN and MCM2-C lacking the NLS were localized in the cytoplasm (Figure 6A). In cells transfected with *gp70* plus *Mcm2-FL* or *gp70* plus mutated *Mcm2*, MCM2-FL and all the MCM2 deletion mutants were detected in the cytoplasm (Figure 6B). These results indicate that gp70 binding inhibits the nuclear translocation of MCM2 and show that MCM2 lacking an NLS remains in the cytoplasm. We confirmed that overexpression of gp70 and/or MCM2-FL or the mutants did not cause any significant changes in the cell-cycle profile of the transfected cells (Figure S5). Furthermore, the transfected gp70 induced the cytoplasmic localization of DNA-PK as well as MCM2 (Figure S6).

MCM2 has 2 NLS domains, NLS1 and NLS2. NLS2 but not NLS1 is required for the nuclear localization of mouse MCM2 [47]. Thus, to further examine the gp70-mediated inhibition of MCM2 nuclear translocation, we generated plasmids encoding HA-tagged MCM2 NLS deletions; deletion of NLS1 (MCM2-ΔNLS1), deletion of NLS2 (MCM2-ΔNLS1), and deletion of NLS1 to NLS2 (MCM2-ΔNLS1-2) (Figure 6C). 3T3 cells were transfected with these mutants and treated with doxorubicin, and apoptotic cell ratios were determined. The ratio was significantly increased in *Mcm2-ΔNLS2*- and *Mcm2-ΔNLS1-2*-transfected cells compared to the negative control. By contrast, *Mcm2-ΔNLS1*-transfected cells exhibited no increase in the number of apoptotic cells (Figure 6D). Furthermore, MCM2-ΔNLS1 was localized in the nucleus, whereas MCM2-ΔNLS2 and MCM2-ΔNLS1-2 were detected in the cytoplasm (Figure S7). These results indicate that deletion of NLS2 alters the subcellular localization of MCM2 and the apoptosis enhancement seen in the presence of the gp70-MCM2.

### Induction of Leukemia cell Apoptosis by DNA-damage in FLV-infected Hosts

**Figure 7:**
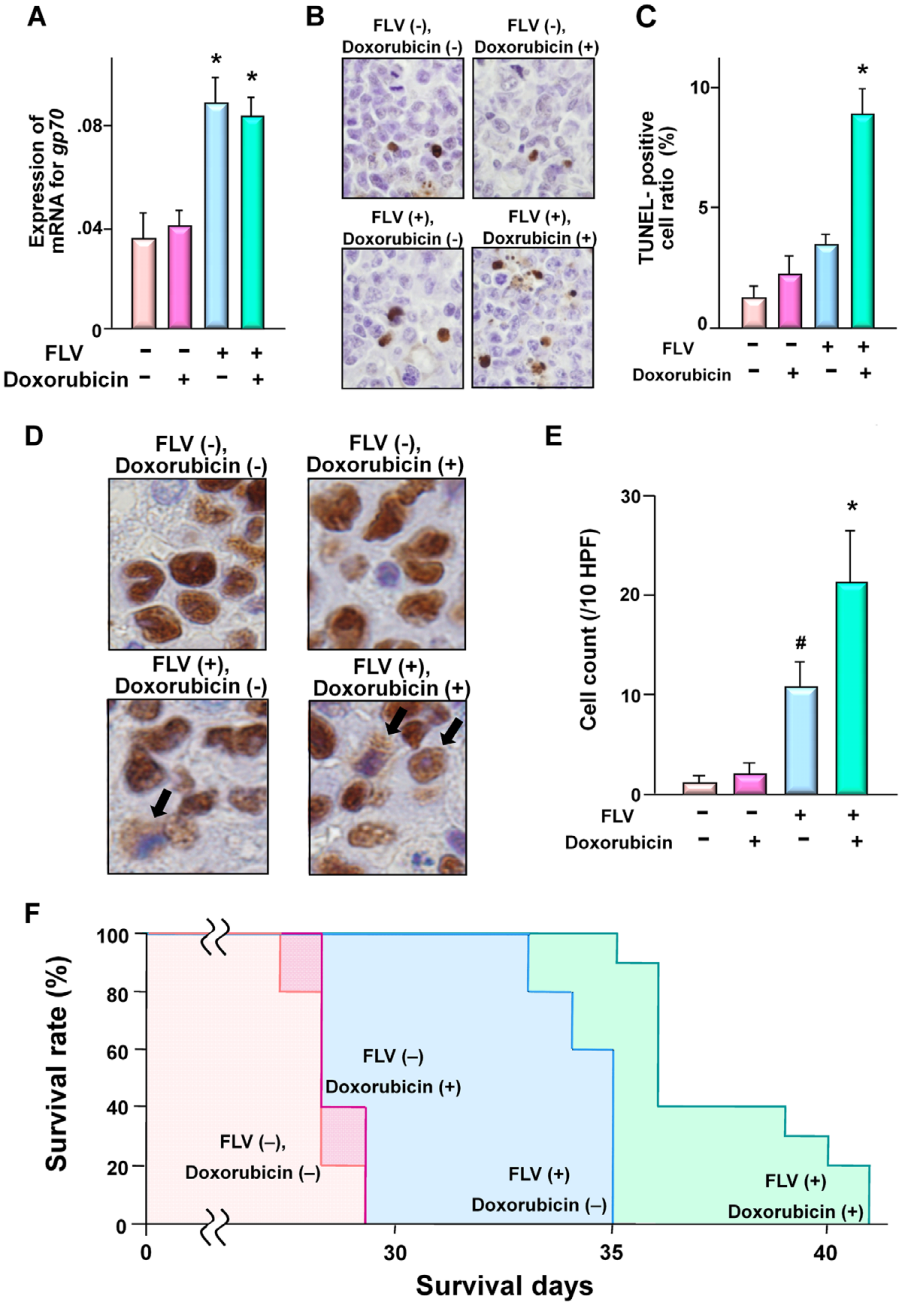
*In vivo* anti-tumor effects of *gp70* expression and DNA-damage on the C3H-derived cells in SCID mice. Two weeks after transplantation, mice were inoculated (i.p.) with FLV. Seven days later, the mice were treated with 1.5 mg/kg of doxorubicin or PBS. (**A**) Quantitative RT-PCR analysis of *gp70* mRNA expression in the liver of SCID mice with multiple foci of leukemic infiltration. The samples from FLV-infected mice exhibit higher levels of *gp70* than those from uninfected mice (*p<0.01). Data represent the mean and 95% CI of from 10 mice in each group and are representative of 2 independent experiments. (**B**) Microscopic features of TUNEL-positive cells in hepatic nodules and (**C**) TUNEL-positive cell ratio in each group of mice. Note the significant increase in apoptotic 8047 cells in mice with FLV infection and doxorubicin treatment (*p<0.01 compared with the tumor cells of “FLV (−), doxorubicin (−) mice”). Data represent the mean and 95% CI of from 10 mice in each group and are representative of 2 independent experiments. (**D**) Subcellular localization of MCM2 in 8047 cells of the liver demonstrated by immunohistochemistry. Images were captured with a microscope at 1,000× magnification power. Note the nuclear and/or cytoplasmic localization of MCM2 in the 8047 cells from each group of mice. (**E**) The cell counts for cytoplasmic localization of MCM2. Cell counts are shown as the number of cells per 10 high-power fields (HPF). [# *p*<0.01 compared with tumor cells of “FLV (−), doxorubicin (−)” mice; *p<0.001 compared with “FLV (−) doxorubicin (−)” mice and *p*<0.05 compared with “FLV (+), doxorubicin (−)” mice]. Data represent the mean and 95% CI of from 10 mice in each group and are representative of 2 independent experiments. (**F**) Kaplan-Meier survival curves for 8047-transplanted SCID mice with/without FLV-infection and doxorubicin-treatment. Note the significant elongation of survival time in mice with FLV-infection [*p*<0.01 compared with “FLV (−), doxorubicin (−)” and “FLV (−), doxorubicin (+)” mice] and in mice with FLV-infection and doxorubicin-treatment [*p*<0.001 compared with “FLV (−), doxorubicin (−)” and “FLV (−), doxorubicin (+)” mice, *p*<0.01 compared with “FLV (+), doxorubicin (−)” mice]. The survival curves represent data from 10 mice in each group.

To determine whether C3H-derived leukemia cells exhibited enhanced apoptosis in response to gp70 and DNA-damage *in vivo*, SCID mice were intravenously transplanted with 8047 cells, inoculated with FLV, and treated with doxorubicin. As expected, the 8047 cell-containing liver samples from FLV-infected mice exhibited a stronger expression of *gp70* than those from uninfected mice (Figure 7A). Treatment with a low dose of doxorubicin caused significant enhancement of apoptosis in FLV-infected SCID mice but not in uninfected mice (Figure 7B, C). These results indicate that *gp70* overexpression and DNA-damage induction elicit significant apoptosis of C3H-derived leukemia cells *in vivo*.

Next, to investigate the subcellular localization of MCM2 in the transplanted 8047 cells from hepatic nodules, immunohistochemistry was performed. MCM2 was localized in the nucleus of 8047 cells in uninfected SCID mice (Figure 7D, top), whereas some 8047 cells exhibited cytoplasmic MCM2 in the FLV-infected mice (Figure 7D, bottom). Furthermore, the number of cells with cytoplasmic MCM2 was remarkably increased in FLV-infected doxorubicin-treated mice compared to FLV-infected PBS-treated mice (Figure 7D, bottom right and E).

A survival analysis was performed on mice treated with PBS or doxorubicin twice a week. FLV-infected and doxorubicin-treated mice exhibited a significant improvement in survival compared to the other groups (Figure 7F). These results suggest significant effects of cytoplasmic MCM2 on apoptosis induction in leukemia cells in the *in vivo* model. Although not so remarkable, FLV-infection alone prolonged the survival of 8047 cell-transplanted mice. The phenomenon may be caused by intrinsic host defense mechanisms such as innate immunity systems and inflammatory reactions by natural killer cells, neutrophils, monocyte/macrophages etc., against leukemia cells. The reactions may include reactive oxygen species or other stress signaling pathways associated with DNA-damage induction. Thus, the circulating leukemia cells may differ from the leukemia cells used *in vitro* experiments without any stimulation for DNA-damage.

## Discussion

A novel strategy for controlling tumor cell growth is to target regulators of cellular proliferation/apoptosis. However, the cellular dynamics of non-tumor cells should not be influenced by these treatments. This is very difficult, but infection with certain types of viruses elicits tumor cell-specific changes in cellular dynamics [48]. Thus, virus-host interaction may provide clues to develop a novel strategy for tumor therapy. Our previous study has shown that FLV infection strongly enhances radiation-induced apoptosis in the hematopoietic cells of C3H mice, although the response is not uniform among the host strains [17]. Elucidation of the molecular mechanisms underlying this host- and cell type-specificity may provide an effective means to induce tumor cell-specific apoptosis in host tissues.

Regarding host specificity, MCM2 was identified as a C3H-specific protein that enhances DNA-damage-induced apoptosis in association with the envelope protein of FLV, gp70. However, MCM2 is part of a conserved set of MCM proteins (MCM2-7), with essential roles in the regulation of DNA replication: functioning as license components for S-phase initiation and further acting as a helicase to unwind DNA at replication forks [25], [26], [49]. Indeed, MCM proteins are frequently overexpressed in a variety of cancer or pre-cancerous cells [31]–[36]. In this study, *Mcm2*-transfected 3T3 cells exhibited an increase in proliferation 96 h after transfection. On the other hand, co-transfection of BALB/c-derived 3T3 cells, which originally expressed low levels of *Mcm2*, with *gp70* and *Mcm2* enhanced doxorubicin-induced apoptosis. These results suggest that human tumor cells may also become more sensitive to DNA-damage-induced apoptosis through changes in the molecular functions of MCM2.

**Figure 8:**
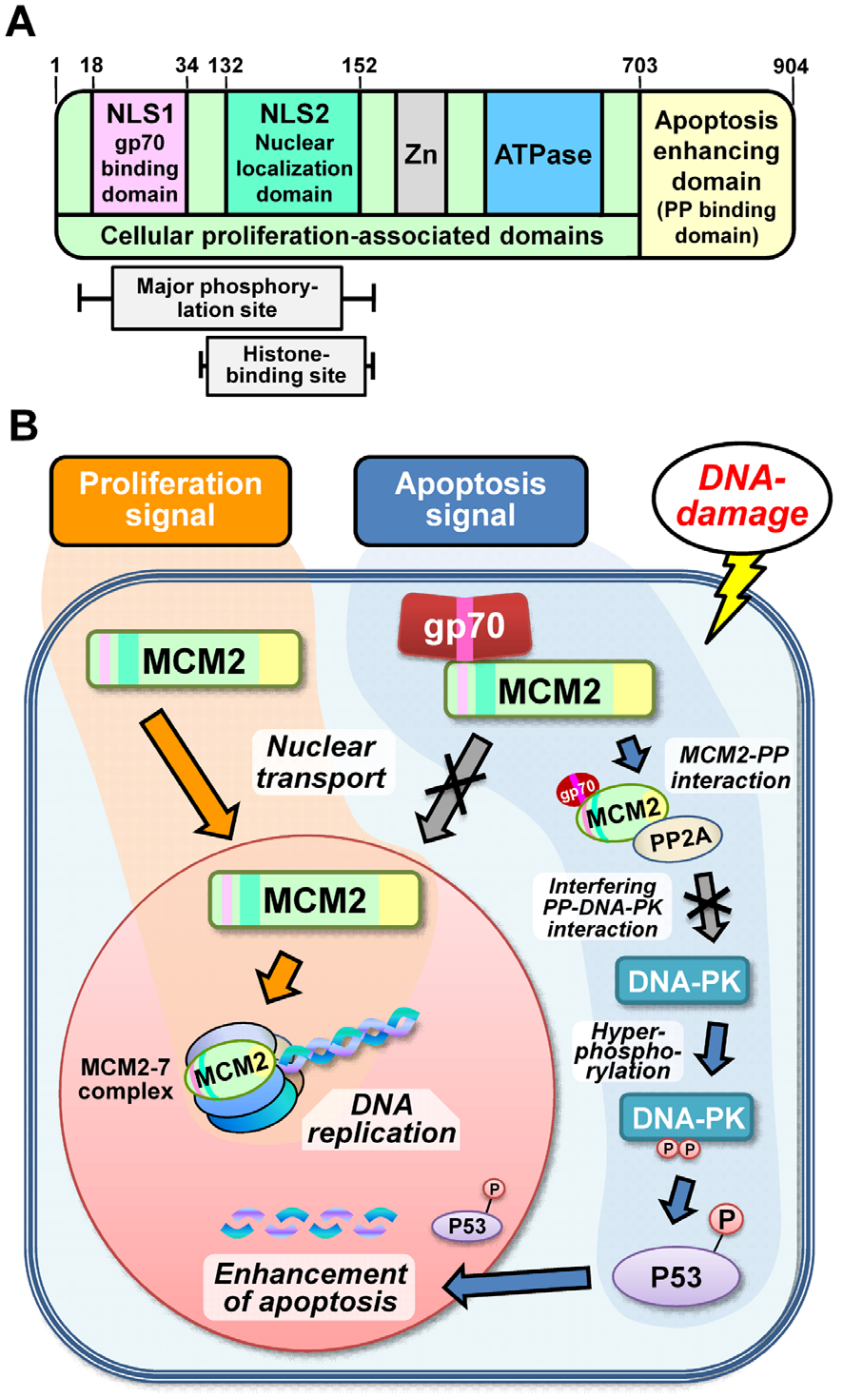
Schematic illustration of the structure of MCM2 and its functions in the cytoplasm and nucleus. (**A**) The various functional domains of the MCM2 protein are shown, and the domains and regions required for the activities are indicated. (**B**) Schematic of the novel role of MCM2 in apoptosis enhancement. Normally, MCM2 is recruited into the nucleus for participation in DNA replication. As a result, cellular proliferation is upregulated (proliferation signal). However, when gp70 is present in the cytoplasm, it binds to MCM2 and inhibits its nuclear entry. Furthermore, cytoplasmic gp70-MCM2-complex interacts with PP2A and inhibits its interaction with DNA-PK. Consequently, hyperphosphorylated DNA-PK enhances DNA-damage-induced apoptosis via a P53-related pathway (apoptosis signal).

MCM2 has several functional domains [50]. However, there are no reports on its functions in apoptosis. Our study demonstrated that a novel functional domain in the C-terminal portion of MCM2 plays a role in apoptosis enhancement under specific conditions in conjunction with gp70 (Figure 8A).

MCM2 is known to interact with various types of molecules, including protein PP2A [51]. PP2A is one of the major Ser/Thr phosphatases implicated in the regulation of cellular processes such as cell cycle progression [52], apoptotic cell death [53]–[55], and DNA replication and DSB repair [45], [52], [53]. In the GeneChip assay of the present study, *Ppp2ac* exhibited an expression pattern similar to that of *Mcm2* in the *in vivo* experiments (correlation coefficient >90%; Figure 1L, Table S1). Furthermore, our results suggest that PP2A dephosphorylates DNA-PK and regulates its function, as described previously [44]–[46]. Depletion of PP2A by RNAi has been shown to induce hyperphosphorylation of DNA-PK and suppression of DNA end-joining followed by enhanced cytogenetic abnormalities including chromosomal and chromatid breaks [46]. Similar events may result from the interaction of PP2A with MCM2.

The MCM complex (MCM2-7) contains an NLS. MCM2 has 2 NLS domains and histone-binding sites in the N-terminal portion, and therefore deletion of the N-terminal portion resulted in the inhibition of nuclear translocation. NLS2 but not NLS1 is required for the nuclear localization of mouse MCM2 [47]. In the present study, nuclear translocation of MCM2 was inhibited by the binding of gp70 to NLS1, and that the cytoplasmic MCM2 enhanced DNA-damage-induced apoptosis.

In conclusion, we identified a novel function of MCM2: the enhancement of DNA-damage-induced apoptosis. This function occurred in association with gp70, an FLV-derived envelope protein. Gp70 directly bound to the N-terminal portion of MCM2 and inhibited its translocation. The cytoplasmic MCM2-gp70-complex induced an interaction of MCM2 with PP2A, thereby interfering with the PP2A-DNA-PK interaction and leading to enhanced DNA-damage-induced apoptosis via the activation of P53 by DNA-PK (Figure 8B). These results suggest that regulation of the molecular dynamics of MCM2 may be a novel apoptosis-inducing therapeutic method to specifically target malignant tumors that express higher levels of MCM2 than normal tissues.

## Materials and Methods

### Ethics Statement

Animal experiments were conducted and carried out in strict accordance with the Act on Welfare and Management of Animals of the government of Japan and the Guidelines for the Care and Use of Laboratory Animals of the Tokyo Medical and Dental University. All experiments were approved by the Animal Experiment Committee of the Tokyo Medical and Dental University (No. 100115). All efforts were made to minimize suffering in animal experiments.

### Mice and Cell Lines

Eight to 10-week-old male C3H/HeJ mice (*H*−*2^k^*) raised under specific-pathogen-free conditions were purchased from Japan SLC, Inc. (Shizuoka, Japan) with the permission of Dr. Yoshiya Shimada of the National Institute of Radiological Sciences in Chiba. Specific-pathogen-free C57BL/6J mice (*H−2^b^*) and BALB/c mice (*H*−*2^d^*) aged 8–10 weeks were also purchased from Japan SLC, Inc. Six-week-old male specific-pathogen-free SCID mice (C.B.17*^scid/scid^*, *H*−*2^d^*) were purchased from CLEA Japan Inc. (Tokyo, Japan).

The mouse fibroblast cell line 3T3 and the mouse acute myeloid leukemia cell line, BaF3, both derived from BALB/c mice, and the C3H mouse bone marrow cell-derived 32D cells were purchased from the RIKEN Cell Bank (Tsukuba, Ibaraki, Japan). The radiation-induced myeloid leukemia cell line from C3H mice, 8047, was established at the National Institute of Radiological Sciences in Chiba [22]. The cells were cultured in RPMI-1640 medium (Sigma, St. Louis, MO, USA). Primary cultured fibroblasts were derived from the lungs of BALB/c and C3H mice and cultured in DMEM (Sigma). The medium was supplemented with 10% fetal calf serum (FCS), penicillin (50 units/mL) (Invitrogen, Carlsbad, CA, USA), and streptomycin (50 µg/mL) (Invitrogen) and the cells were cultured at 37°C in a humidified atmosphere of 5% CO_2_ in air.

### Antibodies and Reagents

Rabbit polyclonal anti-glyceraldehyde-3-phosphate dehydrogenase (GAPDH) antibody (Santa Cruz Biotechnology, Santa Cruz, CA, USA), rabbit polyclonal anti-ATM antibody (MILLIPORE, Billerica, MA, USA), mouse monoclonal anti-DNA-PK_cs_ antibody (Santa Cruz), rabbit polyclonal anti-DNA-PK S2056 (Mouse-S2053) antibody (Assay Biotech, Sunnyvale, CA, USA), mouse monoclonal anti-P53 antibody (Merck, Darmstadt, Germany), rabbit polyclonal anti-phospho-P53 (Ser 15) antibody (Merck), rabbit monoclonal anti-cleaved caspase-3 antibody (Cell Signaling Technology [CST], Danvers, MA, USA), rabbit polyclonal anti-MCM3 antibody (CST), mouse monoclonal anti-MCM4 antibody (Santa Cruz Biotechnology), mouse monoclonal anti-HA tag antibody (Invivogen, San Diego, CA, USA), and mouse monoclonal anti-FLAG M2 antibody (Sigma) were used as primary antibodies for immunoblotting. Rabbit polyclonal anti-FLAG antibody (Sigma), rabbit polyclonal anti-HA antibody (Sigma), and rabbit polyclonal anti-PP2A antibody (CST) were used for immunoprecipitation. Horseradish peroxidase (HRP)-conjugated anti-mouse IgG and HRP-conjugated anti-rabbit IgG (GE Healthcare, Little Chalfont Buckinghamshire, England) were used as secondary antibodies for immunoblotting. Doxorubicin hydrochloride (Wako, Tokyo, Japan) was used for DNA-damage induction. NU7026 (Calbiochem, La Jolla, CA, USA) was used to inhibit DNA-PK activity. Okadaic acid (OA; Wako) was used to inhibit PP2A.

### Viral Infection and DNA-damage Induction

The NB-tropic FLV complex, originally provided by Dr. C. Friend, was prepared as described previously [56]. Eight- to 10-week-old BALB/c, C57BL/6, and C3H mice were inoculated intraperitoneally (i.p.) with FLV at a highly leukemogenic dose of 10^4^ PFU/mouse [57]. On day 7 after the infection with FLV, BALB/c, C57BL/6, and C3H mice were administered (i.p.) with 1.5 mg/kg of doxorubicin hydrochloride. In experiments *in vitro*, 3T3 cells were treated with 1 µM doxorubicin to induce apoptosis.

### Detection of Apoptotic Cells

To determine the apoptotic cell ratios in mouse bone marrow and spleen cells after treatment with 1.5 mg/kg of doxorubicin for 24 h, samples were collected from each experimental group, washed with ice-cold PBS, stained with propidium iodide (BD Biosciences, San Jose, CA, USA) and fluorescein isothiocyanate (FITC)-labeled anti-annexin V antibody (BD), and analyzed on a FACScan flow cytometer (BD FACSCanto™ Flow Cytometer). To determine the apoptotic cell ratios in 32D, BaF3, and 3T3 cells after treatment with 1 µM doxorubicin for 24 h, samples were collected from each experimental group and washed with ice-cold PBS. These samples were stained with propidium iodide (PI), incubated with FITC-labeled anti-annexin V antibody, and analyzed on a FACScan flow cytometer. For detecting apoptotic cells in tissue sections, the terminal deoxy-transferase (TdT)-mediated dUTP nick-end labeling (TUNEL) method was used as previously described [58]. Briefly, tissue sections were deparaffinized and incubated with proteinase K (prediluted, DAKO Cytomation, Glostrup, Denmark) for 15 min at room temperature. After the tissues were washed, TdT, FITC-dUTP and -dATP (BoehringerMannheim, Mannheim, Germany) were applied and the sections were incubated in a moist chamber for 60 min at 37°C. Anti-FITC-conjugated antibody-peroxidase (POD converter, Boehringer Mannheim) was employed to detect FITC-dUTP labeling, and color development was achieved with 3,3′-diaminobenzidine (DAB) solution containing 0.3% hydrogen peroxide. The sections were then observed under a microscope and the proportion of TUNEL-positive cells was determined by dividing the number of positively stained cells by the total number of cells.

### Sybr Green Real-time RT-PCR

RNA was extracted from the bone marrow and spleen cells of BALB/c, C57BL/6, and C3H mice, 8047, 32D, BaF3, and 3T3 cell lines, and primary cultured fibroblasts using Trizol (Invitrogen) according to the manufacturer’s instructions. Briefly, the liquid phase was incubated with chloroform for phase separation. Total RNA was finally extracted using one isopropanol precipitation step and one ethanol wash. The RNA pellet was diluted in RNase- and DNase- free water (Qiagen, Hilden, Germany). Then cDNA was generated from RNA using TaqMan® Reverse Transcription Reagents (Applied Biosystems, Foster, CA, USA) and quantitative RT-PCR was performed. For quantitative RT-PCR, specific primers were used with the Lightcycler Sybr Green master mix (Roche, Basel, Switzerland). The sequences of the primers are as follows: for *Mcm2*, GAGGATGGAGAGGAACTCATTG and ATCTTCCTCGCTGCTGTCA; for *Dna-pk*, GAATTCACCACAACCCTGCT and GCTTTCAGCAGGTTCACACA; for *Atm*, CCTTTGTCCTTCGCGATGTTA and GCTGTATGACAAACTCGACTTAATAGGT; and for *gp70*, AAGGTCCAGCGTTCTCAAAAC and AGGTGGCGTTAGCTGTTTGT. The PCR product was detected using the ABI Prism 7900HT Sequence Detection System (Applied Biosystems [ABI], Carlsbad, CA, USA). The primers and TaqMan probes for *Gapdh* were purchased from ABI. *Mcm2*, *Dna-pk*, *Atm,* and *gp70* RNA levels were normalized to that of *Gapdh*.

### GeneChip Analysis

FLV-infected or uninfected C57BL/6 and C3H mice were administered (i.p.) with 15 mg/kg (high dose) or 1.5 mg/kg (low dose) of doxorubicin, and the spleen was sampled at 0, 1, 6, and 24 h after administration. Total RNA was isolated using RNeasy mini kit (Qiagen), according to the manufacturer’s instructions. First-strand cDNAs were synthesized by incubating 5 µg of total RNA with 200 U of SuperScript II reverse transcriptase (Invitrogen) and 100 pmol of the T7-(dT)_24_ primer [5′-GGCCAGTGAATTGTAATACGACTCACTATAGGGAGGCGG-(dT)_24_-3′]. After second-strand synthesis, the double-stranded cDNAs were purified using a GeneChip Sample Cleanup Module (Affymetrix, Santa Clara, CA, USA), according to the manufacturer’s instructions. Double-stranded cDNAs were labeled by *in vitro* transcription using a BioArray High Yield RNA transcript labeling kit (Enzo Diagnostics, Farmingdale, NY, USA). The labeled cRNA was then purified using a GeneChip Sample Cleanup Module (Affymetrix) and treated with 1× fragmentation buffer (40 mM acetate, 100 mM KOAc, 30 mM MgOAc) at 94°C for 35 min. For hybridization to a GeneChip Mouse Genome 430 2.0 Array (Affymetrix), 15 µg of fragmented cRNA probe was incubated with 50 pM control oligonucleotide B2, 1× eukaryotic hybridization control (1.5 pM BioB, 5 pM BioC, 25 pM BioD and 100 pM Cre), 0.1 mg/mL herring sperm DNA, 0.5 mg/mL acetylated BSA and 1× manufacturer-recommended hybridization buffer in a 45°C rotisserie oven for 16 h. Washing and staining were performed with GeneChip Fluidic Station (Affymetrix) using the appropriate antibody dilution, washing and staining protocol. The phycoerythrin-stained arrays were scanned as digital image files and the scanned data were analyzed with GeneChip Operating Software (Affymetrix) [59]. All data are available online (http://www.nihs.go.jp/tox/TtgSubmitted.htm) from the National Institute of Health Sciences.

### Transfection of Expression Plasmids

Sequences of full-length mouse MCM2 (MCM2-FL) and MCM2 deletion mutants, MCM2-ΔC (amino acid [aa] 1–703), MCM2-ΔN (aa 156–703), MCM2-N (aa 1–155) and MCM2-C (aa 704–904), were amplified from the cDNA of 8047 cells using PCR primers, and inserted into the *Hind*III/*Xho*I site of the *pcDNA™3.1 3*×*HA* Expression Vector (Invitrogen). The primers, synthesized at a commercial laboratory (Invitrogen), were as follows: for *Mcm2-FL*, the 5′ primer was GCTCGAGGCGCGGAGTCTTCTGAGTCTCTCTCA and the 3′ primer was ATAAGCTTTCAGAACTGCTGTAGGATCAG; for *Mcm2-ΔC*, GCTCGAGGCGCGGAGTCTTCTGAGTCTCTCTCA and ATAAGCTTTCACTCCAAGGTGCCACCATTA; for *Mcm2-ΔN*, GCTCGAGGCCGCCACAGAGGATGGCGAGGA and ATAAGCTTTCAGAACTGCTGTAGGATCAG; for *Mcm2-N*, GCTCGAGGCGCGGAGTCTTCTGAGTCTCTCTCA and ATAAGCTTTCAGCGTTCTACGTGGCGGCGC; and for *Mcm2-C*, GCTCGAGGCCCAGCCATGCCCAACACATAT and ATAAGCTTTCAGAACTGCTGTAGGATCAG. The sequence encoding viral gp70 protein was amplified from the cDNA of FLV-infected 8047 cells using the PCR primers, and inserted into the *Not*I/*Xba*I site of the *p3*×*FLAG-CMV™ -10* Expression Vector (Sigma). The primers for *gp70* were ATAAGAATGCGGCCGCGAAAGGTCCAGCGTTCTCAAAA and GCTCTAGACTAGCTAGCTATGCAGCTATGCCGCCCATAGT. The *3*×*HA-Mcm2-ΔNLS1*, *Mcm2-ΔNLS2*, and *Mcm2-ΔNLS1-2* constructs were generated by PCR using a KOD-Plus-Mutagenesis kit (TOYOBO, Tokyo, Japan). The primers, synthesized at a commercial laboratory (Invitrogen), were as follows: for *Mcm2-ΔNLS1*, the 5′ primer was CCGGCGCCGCTGACGGGCAGGGCTA and the 3′ primer was GACGCCCTGACCTCCAGCCCTGGCA; for *Mcm2-ΔNLS2*, GCGCATGCGTCCCAGGCCTCTGCCA and CACGTAGAACGCGCCACAGAGGATG; and for *Mcm2-ΔNLS1-2*, CCGGCGCCGCTGACGGGCAGGGCTA and CACGTAGAACGCGCCACAGAGGATG. The *3*×*HA-Mcm2*, *Mcm2-ΔC*, *Mcm2-ΔN*, *Mcm2-C*, *Mcm2-N*, *Mcm2-ΔNLS1*, *Mcm2-ΔNLS2*, *Mcm2-ΔNLS1-2,* and/or *3*×*FLAG-gp70* constructs were transfected into 3T3 cells (2×10^5^ cells) using Hily Max Transfection Reagent (Nippon Gene, Tokyo, Japan). The controls were generated by mock-transfection with an empty vector.

### Cell Viability Assay

Cell viability was measured using a Cell Proliferation Kit (3-[4,5-dimethylthiazol-2-yl]-2,5-diphenyltetrazolium bromide, MTT) (Roche). Briefly, 3T3 cells were seeded in 96-well plates at 1×10^3^/well. After incubation for 24 h, cells were transfected with 3×*HA-Mcm2*, *Mcm2-ΔC*, *Mcm2-ΔN*, *Mcm2-C*, *Mcm2-N*, and/or *3*×*FLAG-gp70*. Twenty-four hours after the transfection, the cells were treated with 1 µM doxorubicin in culture medium for 24 h. Then 10 µL of MTT labeling reagent was added to each well and incubation continued for 4 h at 37°C. Next, 100 µL of solubilization solution was added to each well and incubation was continued overnight at 37°C. Absorbance was determined at 560 nm using a microplate reader (Bio-Rad, Hercules, CA, USA).

### RNA Interference

Small-interfering RNA (siRNA) was used to silence the *Mcm2* gene. The sequence of siRNA used was CAGGTGACAGACTTTATCAAA. An irrelevant siRNA (GCACACAGACTGCAATCACAGGTTA) that did not lead to specific degradation of any cellular mRNA was used as a negative control. BaF3 and 32D cells (2×10^5^ cells) were transfected with 120 pmol of *Mcm2* or control siRNA using Amaxa® Cell Line Nucleofector® Kit V (LONZA, Basel, Switzerland) according to the manufacturer’s instructions. The oligonucleotides used for cloning short hairpin RNA (shRNA)-encoding sequences targeting DNA-PK and ATM into the pSUPER vector (Oligoengine, Seattle, WA, USA) were as follows: *Sh-Dna-pk*; GATCCCCAGGGCCAAGCTATCATTCTttcaagagaAGAATGATAGCTTGGCCCTTTTTTA; and *sh-Atm*; GATCCCCCATACTAAAGACATTttcaagagaAATGTCTTTGAGTAGTATGTTTTTA. The annealed oligonucleotides were subcloned into *Bgl*II and *Hin*dIII sites. These constructs were transfected into 3T3 cells (2×10^5^ cells) using Hily Max Transfection Reagent (Nippon Gene). The controls were generated by mock-transfected with a *sh-empty* vector.

### Immunoprecipitation and Immunoblotting

Cell lysates were prepared by incubating cell pellets on ice for 30 min in ice-cold lysis buffer containing 10 mM Tris-HCl, pH 7.5, 5 mM EDTA, 1% Nonidet P-40, 0.02% NaN3, 1 mM PMSF, 0.1% aprotinin, 100 µM leupeptin and 100 µM TPCK (Sigma). Cell lysates were incubated with antibody and Protein G Sepharose™ (GE Healthcare). The immunoprecipitates obtained after centrifugation or whole cell lysates were mixed with 2× sodium dodecyl sulfate (SDS) buffer (125 mM Tris-HCl at pH 6.8, 4% SDS, 20% glycerol, 0.01% bromophenol blue, and 10% 2-mercaptoethanol) and boiled for 10 min. The samples were loaded onto a 5–20% or 3–10% gradient polyacrylamide gel (WAKO), and electrophoretically transferred to nitrocellulose membranes (GE Healthcare). The membranes were blocked with 10% skim milk in PBS, incubated with primary antibodies, washed, and incubated with peroxidase-conjugated secondary antibodies. The protein signal was detected using the ECL Plus Western Blotting Detection System (GE Healthcare).

### Chromatin Loading Assay

Chromatin loading of MCM2 was performed as described previously [60]. Briefly, 3T3 cells were harvested using trypsin, and the cell pellets were lysed by incubating in complete cytoskeleton (CSK) buffer (20 mM HEPES, 100 mM NaCl, 3 mM MgCl_2_, 300 mM sucrose, and 0.1% NP-40) for 15 min on ice. Cytoplasmic fractions were obtained as supernatants after low speed centrifugation (3,000×g) at 4°C. Pellets were rinsed with complete CSK buffer for 10 min on ice and recentrifuged to obtain a chromatin-enriched fraction. Pellets were then sonicated for 5 s in CSK buffer and subjected to high-speed centrifugation (16,000×g). The post-sonication supernatant was designated as the chromatin-bound fraction.

### Analysis of Cell Cycle Distribution

Cell cycle distribution was monitored by quantifying the cellular DNA content after staining with PI. Cells were fixed with ethanol for 20 min at −20°C. After centrifugation, cells were suspended in PBS containing PI (50 µg/mL) and RNase (0.2 mg/mL), incubated at room temperature for 30 min, and analyzed on a FACScan flow cytometer (BD FACSCanto™ Flow Cytometer).

### Immunofluorescence

3T3 cells were fixed in 1% paraformaldehyde in PBS and permeabilized with 0.1% NP-40 in PBS at room temperature. Cells were incubated with mouse monoclonal anti-HA antibody (Invivogen) at a 1∶100 dilution in PBS for 1 h at room temperature. Cells were then stained with tetramethylrhodamine-5-(and 6)-isothiocyanate (TRITC)-conjugated anti-rabbit antibody (Dako Cytomation, Glostrup, Denmark) at a 1∶100 dilution for 20 min at room temperature. Slides were washed 3 times with PBS and mounted with Vectashield mounting medium containing 4′,6-diamidino-2-phenylindole (DAPI; Vector Laboratories, Inc., Burlingame, CA, USA). Images were acquired using a BZ-9000 microscope (KEYENCE, Osaka, Japan) with a 400× objective.

### Transplantation of MCM2-expressing Leukemia Cells into SCID Mice and Apoptosis Induction

The 8047 cells (1 x 10^5^ cells) derived from C3H mice were transplanted intravenously into SCID mice via the tail vein. Two weeks after the transplantation, FLV was injected (i.p.) into SCID mice at a dose of 10^4^ PFU/mouse. Then, 7 days after FLV inoculation, the mice were treated twice a week with 1.5 mg/kg of doxorubicin.

### Immunohistochemistry

Formalin-fixed paraffin-embedded tissue sections (4 µm thick) of the liver from 8047-transplanted SCID mice were de-waxed in xylene and re-hydrated through graded alcohol to water. Antigen retrieval was achieved with a 10-min autoclave treatment in 0.1 M citrate buffer (pH6.0). Endogeneous peroxidase activity was inhibited by dipping the slides in 0.3% hydrogen peroxide in methanol for 30 min and non-specific protein binding was blocked by incubation with normal horse serum (Vector Laboratories, Burlingame, CA, USA). Sections were then treated with anti-MCM2 mouse monoclonal antibody (BD Biosciences) (1∶2,000) overnight at 4°C. Detection was achieved using the streptavidin-biotin-peroxidase complex technique (Vector Laboratories) with DAB as the chromogen.

### Statistical Analysis

Statistical significance was determined using a two-tailed Student’s *t*-test. For Kaplan-Meier analysis of SCID mice transplanted with 8047 cells, a log-rank test was performed.

## Supporting Information

Figure S1 **Gp70 suppresses the formation of the MCM complex.** Control, *HA-Mcm2*-transfected and *HA-Mcm2*/*FLAG* **-** *gp70*-transfected 3T3 cells were left untreated or treated with 1 µM doxorubicin for 24 h. Cell lysates were subjected to a pull-down assay to detect the binding of MCM3 or MCM4 to HA-MCM2. In *Mcm2*-transfected 3T3 cells, MCM2 interacts with MCM3 and MCM4, both in the presence and absence of doxorubicin-treatment. By contrast, in *gp70* plus *Mcm2*-transfected 3T3 cells, MCM2 does not co-precipitate with MCM3 or MCM4 after treatment with doxorubicin. These results suggest that gp70 binds to MCM2 and inhibits the formation of the MCM complex and the binding to chromatin under DNA-damage by doxorubicin.
(TIF)

Figure S2 **Gp70 directly interacts with MCM2.** (**A**) Schematic diagram of full-length gp70 (gp70-FL) and the gp70 deletion mutants, gp70-1 (aa 1–153), gp70-2 (aa 154–330), and gp70-3 (aa 331–461). 3T3 cells were transfected with *FLAG*-tagged *gp70* mutants along with *HA*-tagged *Mcm2* and left untreated (**B**) or treated with 1 µM doxorubicin for 24 h (**C**). The expression of the gp70 mutants (**B**, **C**, left upper) and HA-MCM2 (**B**, **C**, left middle) was confirmed in 3T3 cells. Cell lysates were subjected to a pull-down assay to detect the binding of gp70-FL or the mutants to HA-MCM2 (**B**, **C**, right panel). Apoptotic cell ratios were determined with annexin V-staining of *Mcm2-FL*/*gp70* mutant-transfected 3T3 cells that were left untreated (**D**) or treated with 1 µM doxorubicin for 24 h (**E**). Asterisks (*) indicate significant differences between mutant-transfected cells and the control (*p*<0.01). Data represent the mean and 95% CI of 3 independent experiments.
(TIF)

Figure S3 **Effects of MCM2 and deletion mutant overexpression on 3T3 cell proliferation.** 3T3 cells were transfected with *Mcm2-FL* or the *Mcm2* deletion mutants and the cell number was counted at an early phase (48 h, **A**, **B**) and a late phase (96 h, **C**, **D**) after transfection with (**A**, **C**) or without (**B**, **D**) *gp70*. Data represent the mean and 95% CI of 3 independent experiments. Note the significant increase in cell counts following *Mcm2-FL*- and *Mcm2- ΔC*-transfection (*p<0.01).
(TIF)

Figure S4 **Knockdowns of** ***Dna-pk*** **and** ***Atm*** **in** ***gp70*** **plus** ***Mcm2*** **-transfected cells using the pSUPER shRNA system.** The expression of *Dna-pk* (**A**) and *Atm* (**B**) mRNAs and DNA-PK (**C**) and ATM (**D**) proteins were examined by quantitative RT-PCR and western blotting, respectively. Cell survival (**E**, **G**) and apoptotic cell ratio (**F**, **H**) were determined with the MTT assay and annexin V-staining, respectively, after treatment with 1 µM doxorubicin for 24 h. Note the apoptosis-abrogating effects of *sh-Dna-pk* (**E**, **F**). Asterisks (*) indicate significant differences between *sh-Dna-pk*-treated and *sh-Control*-treated cells (*p<0.01). However, *Atm* knockdown causes no remarkable change in viability or apoptotic cell ratio relative to that of cells treated with *sh-Control* (**G**, **H**). Data represent the mean and 95% CI of 3 independent experiments.
(TIF)

Figure S5 **Effects of MCM2 and deletion mutant overexpression on the cell-cycle distribution of 3T3 cells.** 3T3 cells were transfected with the *Mcm2* deletion mutants with (**A**) or without (**B**) *gp70* and treated with 1 µM doxorubicin for 24 h. The cells were fixed with ethanol, stained with propidium iodide (PI), and analyzed by flow cytometry. Data represent the mean and 95% CI of 3 independent experiments. 3T3 cells exhibit an increase in G2/M fraction after treatment with doxorubicin. However, the differences between the cell cycle profiles of *Mcm-2* or *gp70-* transfected cells are not significant.
(TIF)

Figure S6 **Co-localization of gp70, MCM2, and DNA-PK in the cytoplasmic fraction of 3T3 cells.** Control, *HA-Mcm2*-transfected and *HA-Mcm2*/*FLAG* **-** *gp70*-transfected 3T3 cells were left untreated (left) or treated with 1 µM doxorubicin for 24 h (right). Cell lysates from these cells were separated into chromatin-bound and cytoplasmic fractions. HA-MCM2 (upper) and DNA-PK (bottom) were detected by western blotting. In *Mcm2*-transfected 3T3 cells, MCM2 binds to the chromatin irrespective of doxorubicin treatment. By contrast, in *gp70* plus *Mcm2*-transfected 3T3 cells, MCM2 does not bind to the chromatin after treatment with doxorubicin (upper). DNA-PK is not detected in the chromatin-bound and cytoplasmic fractions of samples not treated with doxorubicin. Under doxorubicin-treated conditions, equal proportions of chromatin-bound DNA-PK are seen in all groups. By contrast, DNA-PK is more strongly expressed in the cytoplasmic fraction of *gp70* plus *Mcm2*-transfected 3T3 cells than in the other groups (bottom). These results suggest that gp70, MCM2, and DNA-PK co-localize in the cytoplasm, leading to subsequent P53 activation and apoptosis induction.
(TIF)

Figure S7 **Subcellular localization and interactions of MCM2 NLS deletion mutants in 3T3 cells.** (**A**) 3T3 cells transfected with *HA*-tagged *Mcm2 NLS* deletion mutants were treated with 1 µM doxorubicin for 24 h. The cells were then fixed with 1% paraformaldehyde in PBS, permeabilized with 0.1% NP-40 in PBS at room temperature, and stained with TRITC-conjugated anti-HA antibody. HA-positive cells are shown in red (TRITC), and DAPI-stained nuclei are shown in blue. Images were acquired using a BZ-9000 microscope (KEYENCE) with a 400× objective. Note the nuclear localization of MCM2-ΔNLS1 in contrast to the cytoplasmic localization of MCM2-ΔNLS2 and MCM2-ΔNLS1-2. (**B**) 3T3 cells were transfected with *HA-*tagged *Mcm2 NLS* deletion mutants along with *FLAG-*tagged *gp70*, and treated with 1 µM doxorubicin for 24 h. Expression of the MCM2 NLS deletion mutants (left panel, upper) and FLAG-gp70 (left panel, middle) was confirmed by western blotting. Lysates from these cells were subjected to a pull-down assay to detect the binding of the MCM2 NLS deletion mutants to FLAG-gp70. MCM2-FL and MCM2-ΔNLS2 proteins coprecipitate with gp70 (right panel). Thus, gp70 is able to interact with MCM2-FL and MCM2-ΔNLS2, but not with MCM2-ΔNLS1 or MCM2-ΔNLS1-2. These results suggest that gp70 is bound to the NLS1 domain of MCM2 and indirectly inhibits the function of NLS2.
(TIF)

Table S1 **Identification of genes with expression patterns similar to that of** ***Mcm2*** **using the GeneChip assay.** Gene expression patterns were determined by the GeneChip assay in FLV-infected or un-infected C3H/C57BL/6 mice after treatment with doxorubicin. A part of genes exhibited similar expression patterns with *Mcm2*. The similarity in gene expression patterns was evaluated with the Percellome system using a Pearson product-moment correlation coefficient.
(DOCX)
