# Supplementary figures and images for: Minichromosome Maintenance 2 Bound with Retroviral Gp70 Is Localized to Cytoplasm and Enhances DNA-Damage-Induced Apoptosis
Source: PLoS One. 2012 Jun 29;7(6):e40129. doi: 10.1371/journal.pone.0040129 (PMC3387003; doi:10.1371/journal.pone.0040129)

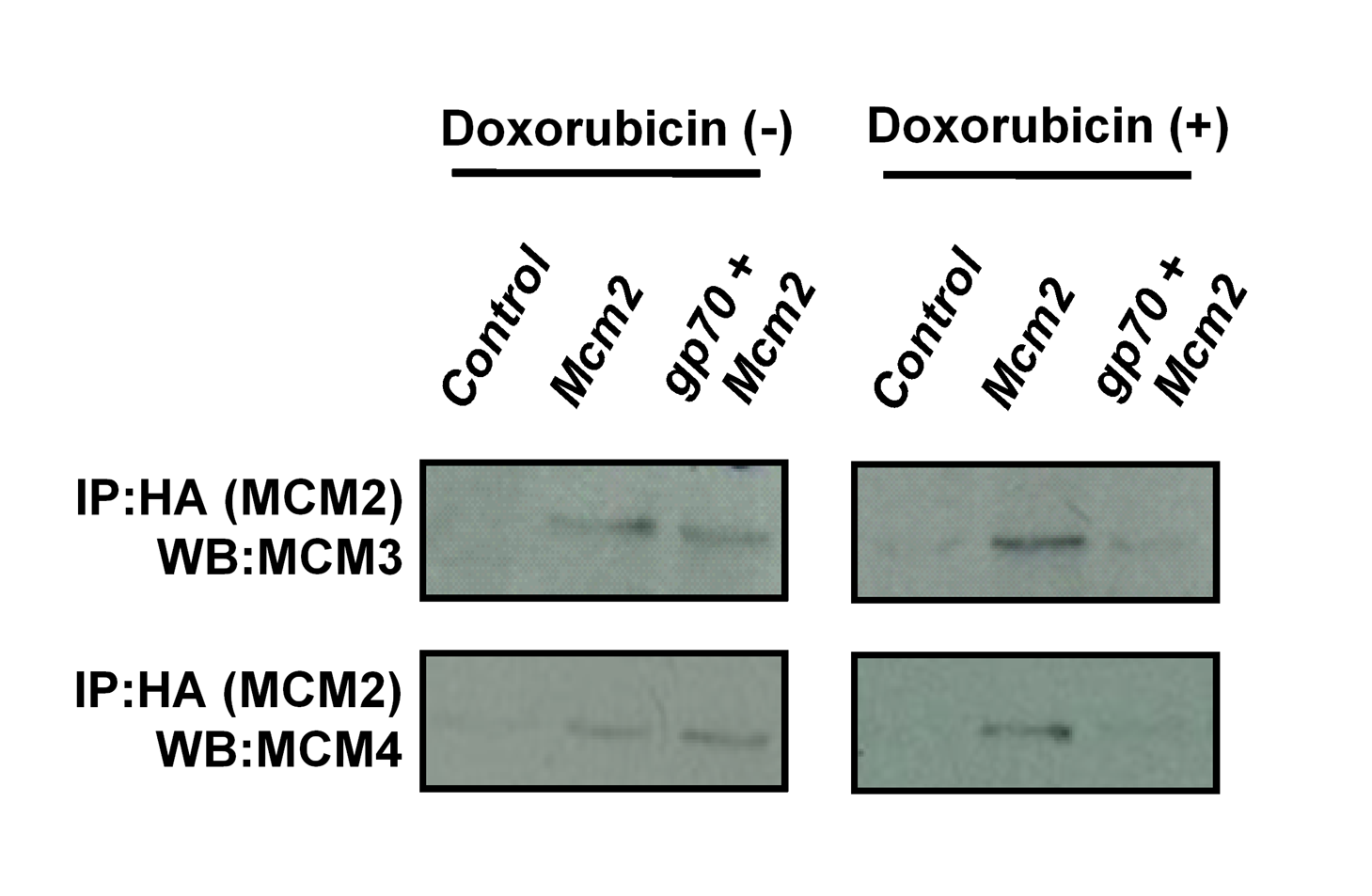

Supplement: Figure S1 — Gp70 suppresses the formation of the MCM complex. Control, HA-Mcm2-transfected and HA-Mcm2/FLAG - gp70-transfected 3T3 cells were left untreated or treated with 1 µM doxorubicin for 24 h. Cell lysates were subjected to a pull-down assay to detect the binding of MCM3 or MCM4 to HA-MCM2. In Mcm2-transfected 3T3 cells, MCM2 interacts with MCM3 and MCM4, both in the presence and absence of doxorubicin-treatment. By contrast, in gp70 plus Mcm2-transfected 3T3 cells, MCM2 does not co-precipitate with MCM3 or MCM4 after treatment with doxorubicin. These results suggest that gp70 binds to MCM2 and inhibits the formation of the MCM complex and the binding to chromatin under DNA-damage by doxorubicin. (TIF) [file pone.0040129.s001.tif]

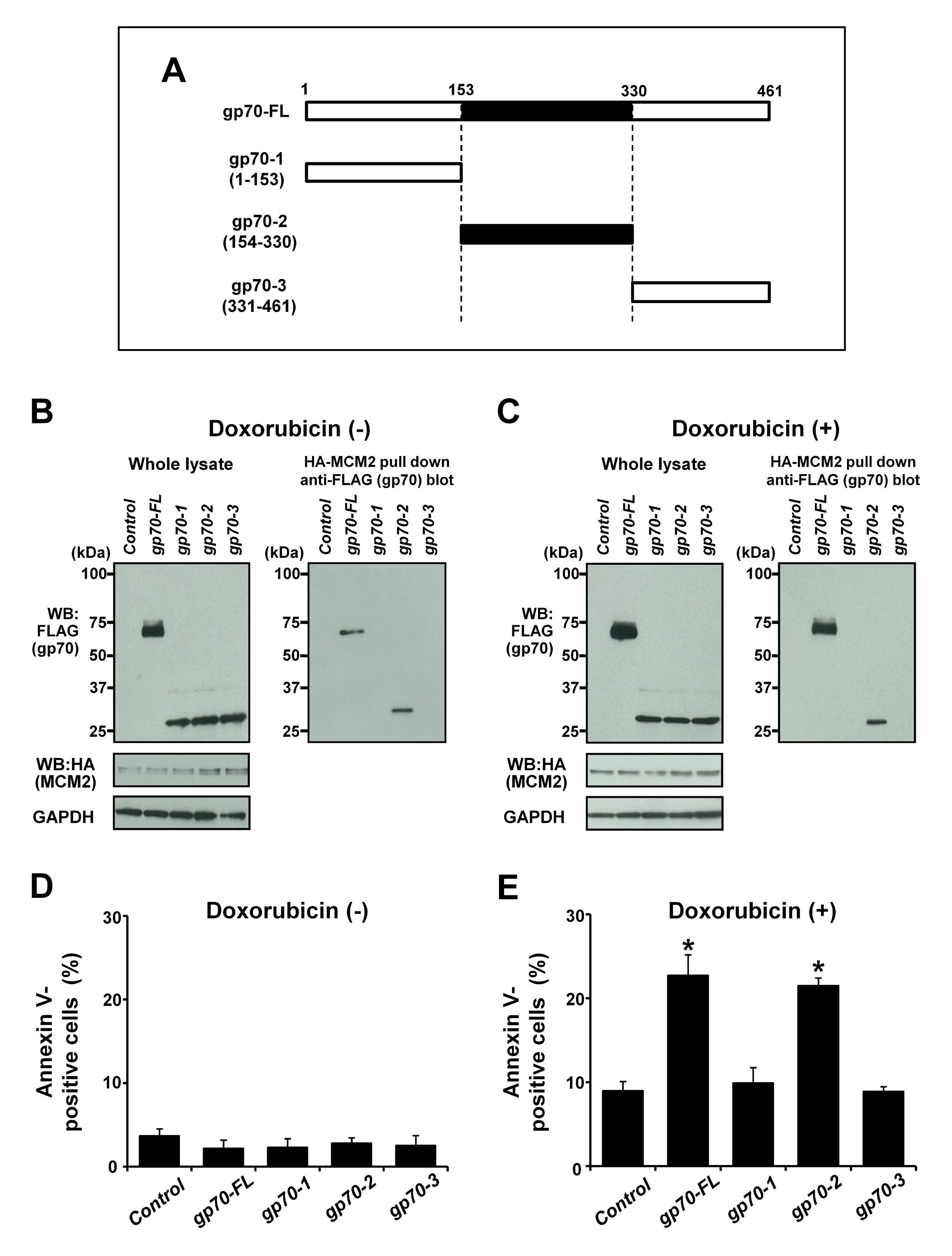

Supplement: Figure S2 — Gp70 directly interacts with MCM2. (A) Schematic diagram of full-length gp70 (gp70-FL) and the gp70 deletion mutants, gp70-1 (aa 1–153), gp70-2 (aa 154–330), and gp70-3 (aa 331–461). 3T3 cells were transfected with FLAG-tagged gp70 mutants along with HA-tagged Mcm2 and left untreated (B) or treated with 1 µM doxorubicin for 24 h (C). The expression of the gp70 mutants (B, C, left upper) and HA-MCM2 (B, C, left middle) was confirmed in 3T3 cells. Cell lysates were subjected to a pull-down assay to detect the binding of gp70-FL or the mutants to HA-MCM2 (B, C, right panel). Apoptotic cell ratios were determined with annexin V-staining of Mcm2-FL/gp70 mutant-transfected 3T3 cells that were left untreated (D) or treated with 1 µM doxorubicin for 24 h (E). Asterisks (*) indicate significant differences between mutant-transfected cells and the control (p<0.01). Data represent the mean and 95% CI of 3 independent experiments. (TIF) [file pone.0040129.s002.tif]

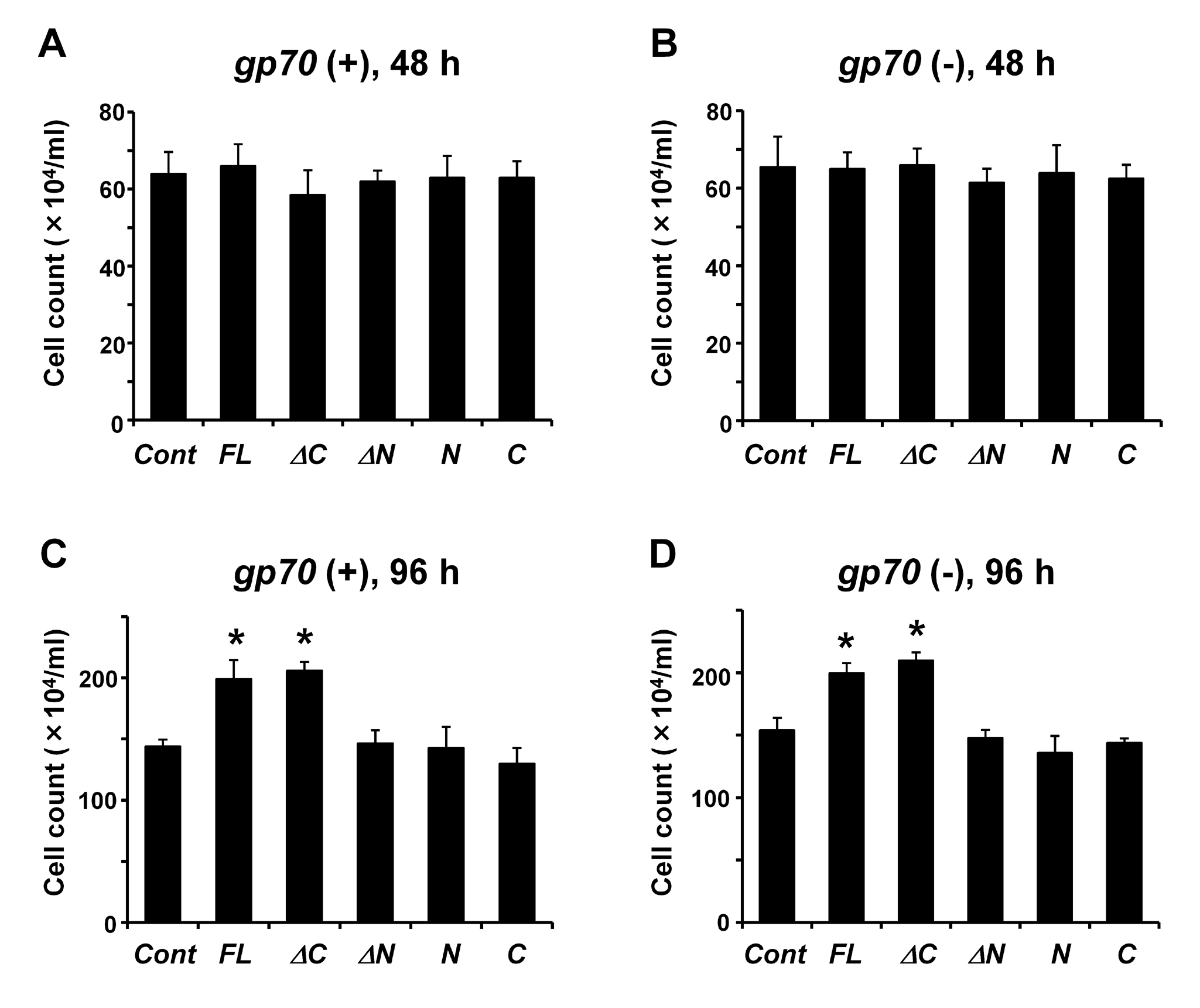

Supplement: Figure S3 — Effects of MCM2 and deletion mutant overexpression on 3T3 cell proliferation. 3T3 cells were transfected with Mcm2-FL or the Mcm2 deletion mutants and the cell number was counted at an early phase (48 h, A, B) and a late phase (96 h, C, D) after transfection with (A, C) or without (B, D) gp70. Data represent the mean and 95% CI of 3 independent experiments. Note the significant increase in cell counts following Mcm2-FL- and Mcm2- ΔC-transfection (*p<0.01). (TIF) [file pone.0040129.s003.tif]

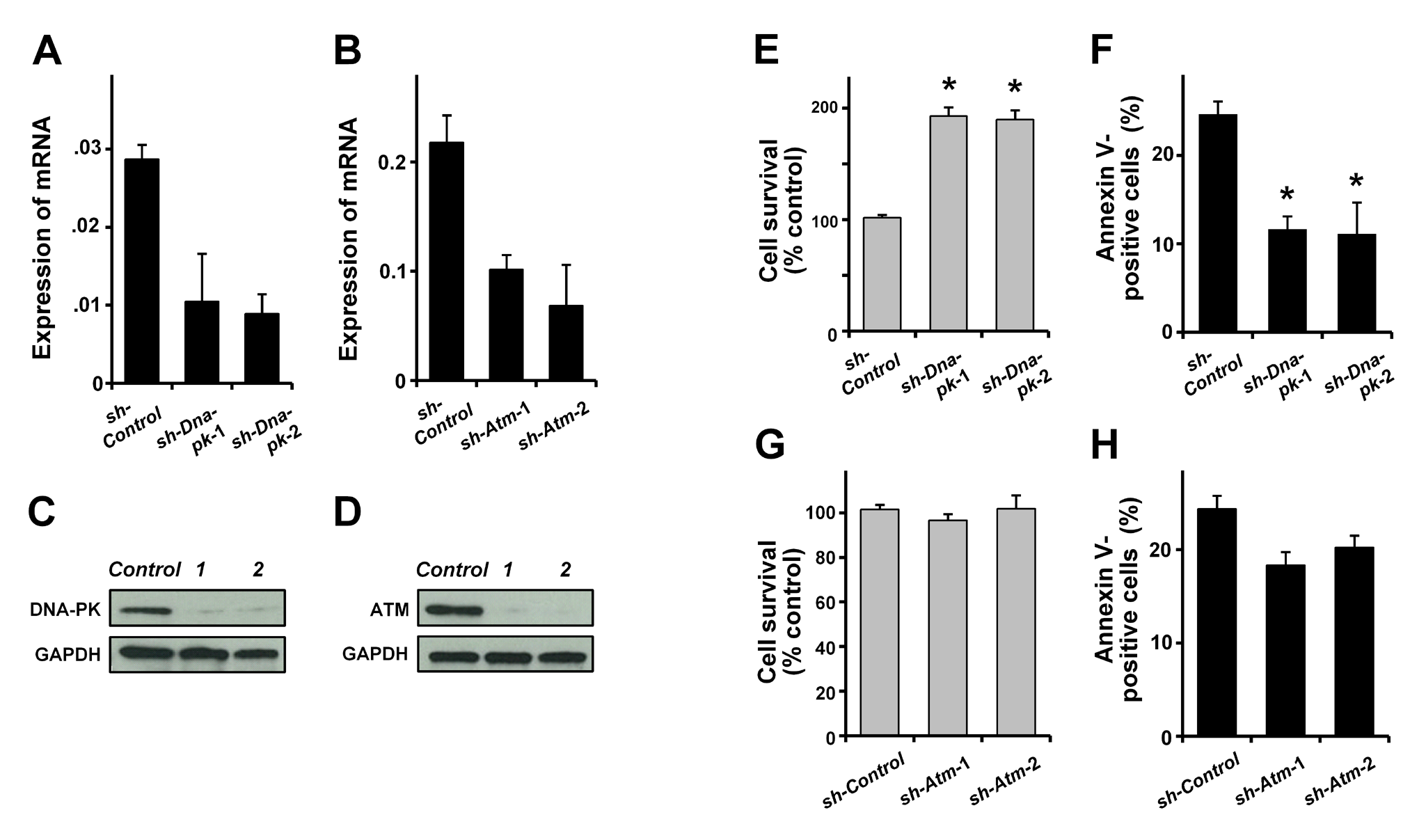

Supplement: Figure S4 — Knockdowns of Dna-pk and Atm in gp70 plus Mcm2 -transfected cells using the pSUPER shRNA system. The expression of Dna-pk (A) and Atm (B) mRNAs and DNA-PK (C) and ATM (D) proteins were examined by quantitative RT-PCR and western blotting, respectively. Cell survival (E, G) and apoptotic cell ratio (F, H) were determined with the MTT assay and annexin V-staining, respectively, after treatment with 1 µM doxorubicin for 24 h. Note the apoptosis-abrogating effects of sh-Dna-pk (E, F). Asterisks (*) indicate significant differences between sh-Dna-pk-treated and sh-Control-treated cells (*p<0.01). However, Atm knockdown causes no remarkable change in viability or apoptotic cell ratio relative to that of cells treated with sh-Control (G, H). Data represent the mean and 95% CI of 3 independent experiments. (TIF) [file pone.0040129.s004.tif]

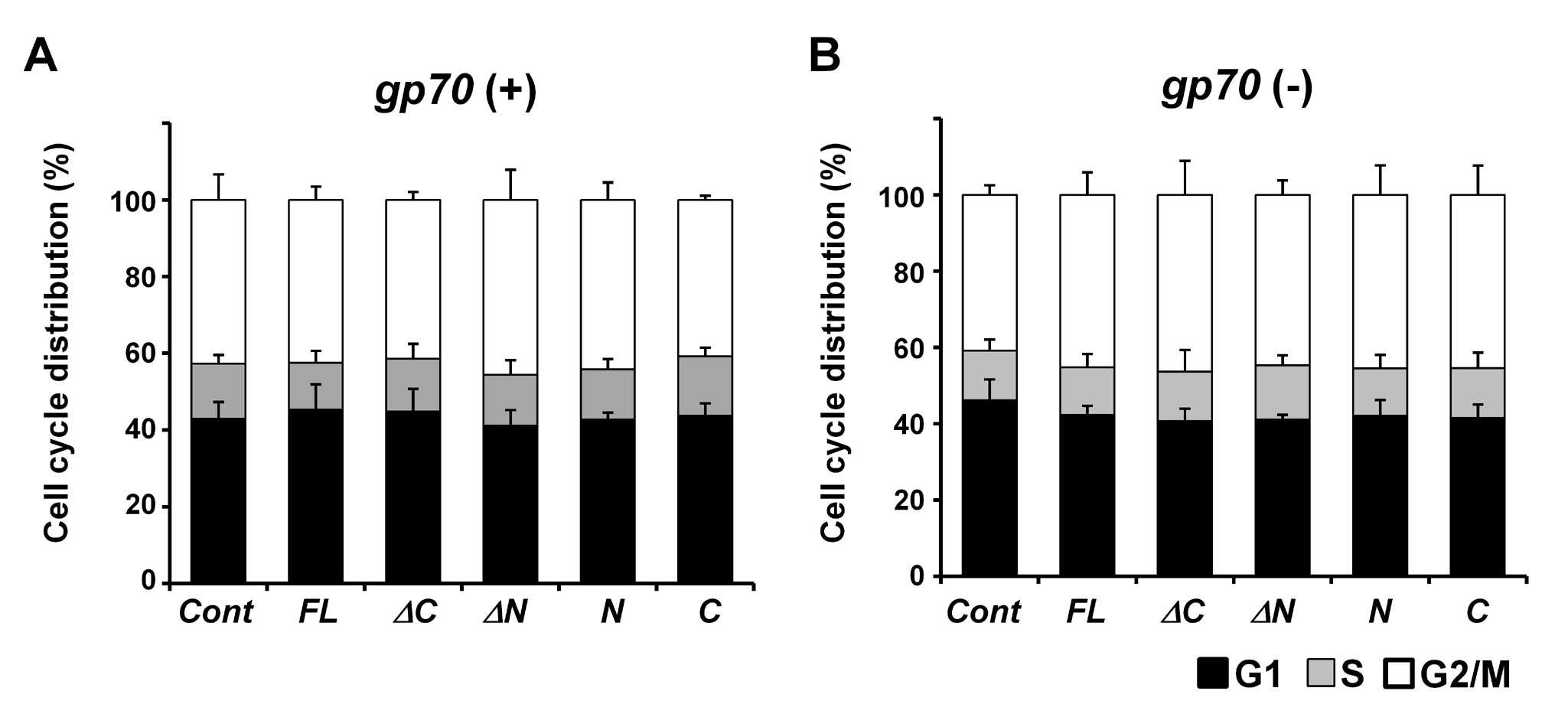

Supplement: Figure S5 — Effects of MCM2 and deletion mutant overexpression on the cell-cycle distribution of 3T3 cells. 3T3 cells were transfected with the Mcm2 deletion mutants with (A) or without (B) gp70 and treated with 1 µM doxorubicin for 24 h. The cells were fixed with ethanol, stained with propidium iodide (PI), and analyzed by flow cytometry. Data represent the mean and 95% CI of 3 independent experiments. 3T3 cells exhibit an increase in G2/M fraction after treatment with doxorubicin. However, the differences between the cell cycle profiles of Mcm-2 or gp70- transfected cells are not significant. (TIF) [file pone.0040129.s005.tif]

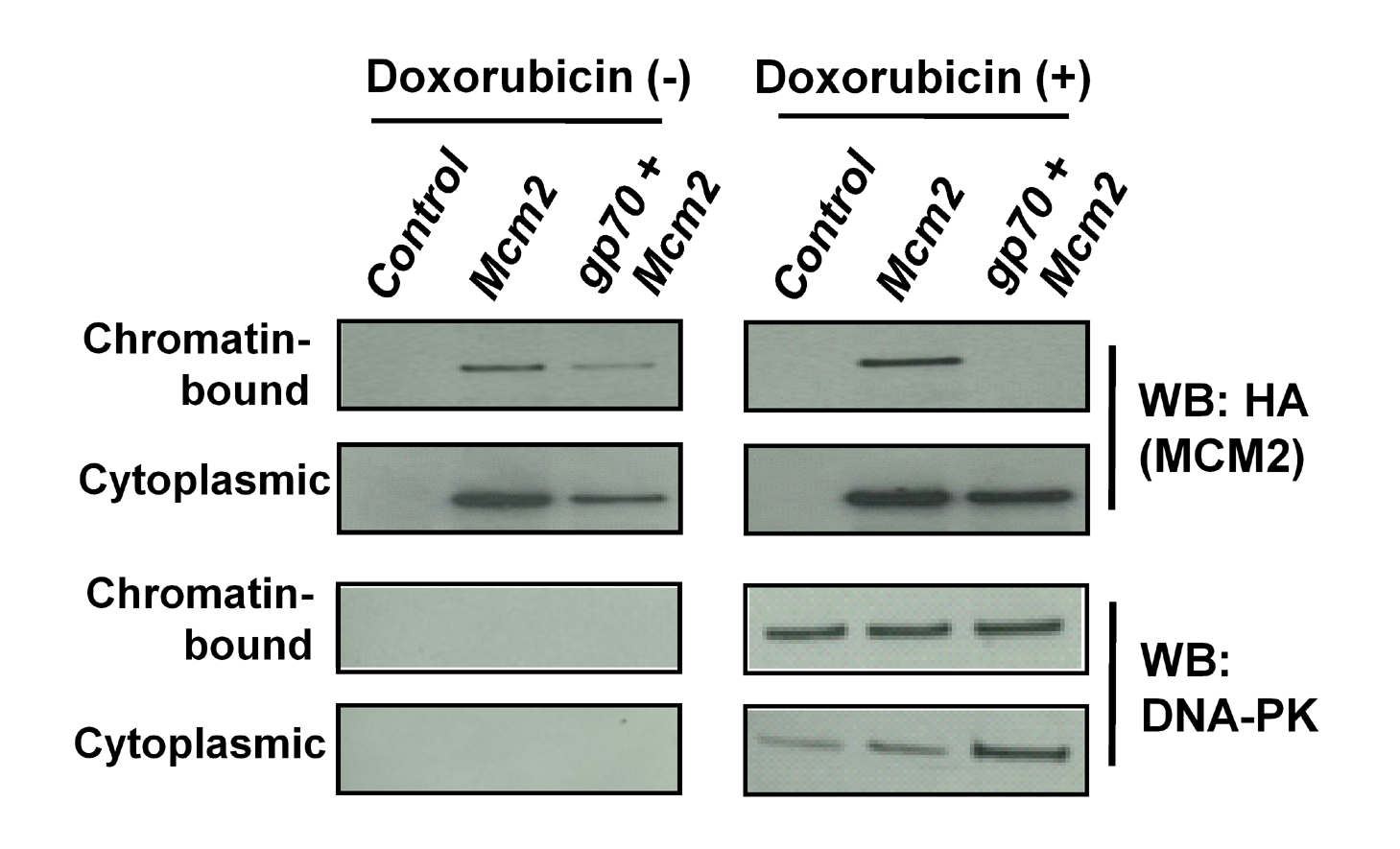

Supplement: Figure S6 — Co-localization of gp70, MCM2, and DNA-PK in the cytoplasmic fraction of 3T3 cells. Control, HA-Mcm2-transfected and HA-Mcm2/FLAG - gp70-transfected 3T3 cells were left untreated (left) or treated with 1 µM doxorubicin for 24 h (right). Cell lysates from these cells were separated into chromatin-bound and cytoplasmic fractions. HA-MCM2 (upper) and DNA-PK (bottom) were detected by western blotting. In Mcm2-transfected 3T3 cells, MCM2 binds to the chromatin irrespective of doxorubicin treatment. By contrast, in gp70 plus Mcm2-transfected 3T3 cells, MCM2 does not bind to the chromatin after treatment with doxorubicin (upper). DNA-PK is not detected in the chromatin-bound and cytoplasmic fractions of samples not treated with doxorubicin. Under doxorubicin-treated conditions, equal proportions of chromatin-bound DNA-PK are seen in all groups. By contrast, DNA-PK is more strongly expressed in the cytoplasmic fraction of gp70 plus Mcm2-transfected 3T3 cells than in the other groups (bottom). These results suggest that gp70, MCM2, and DNA-PK co-localize in the cytoplasm, leading to subsequent P53 activation and apoptosis induction. (TIF) [file pone.0040129.s006.tif]

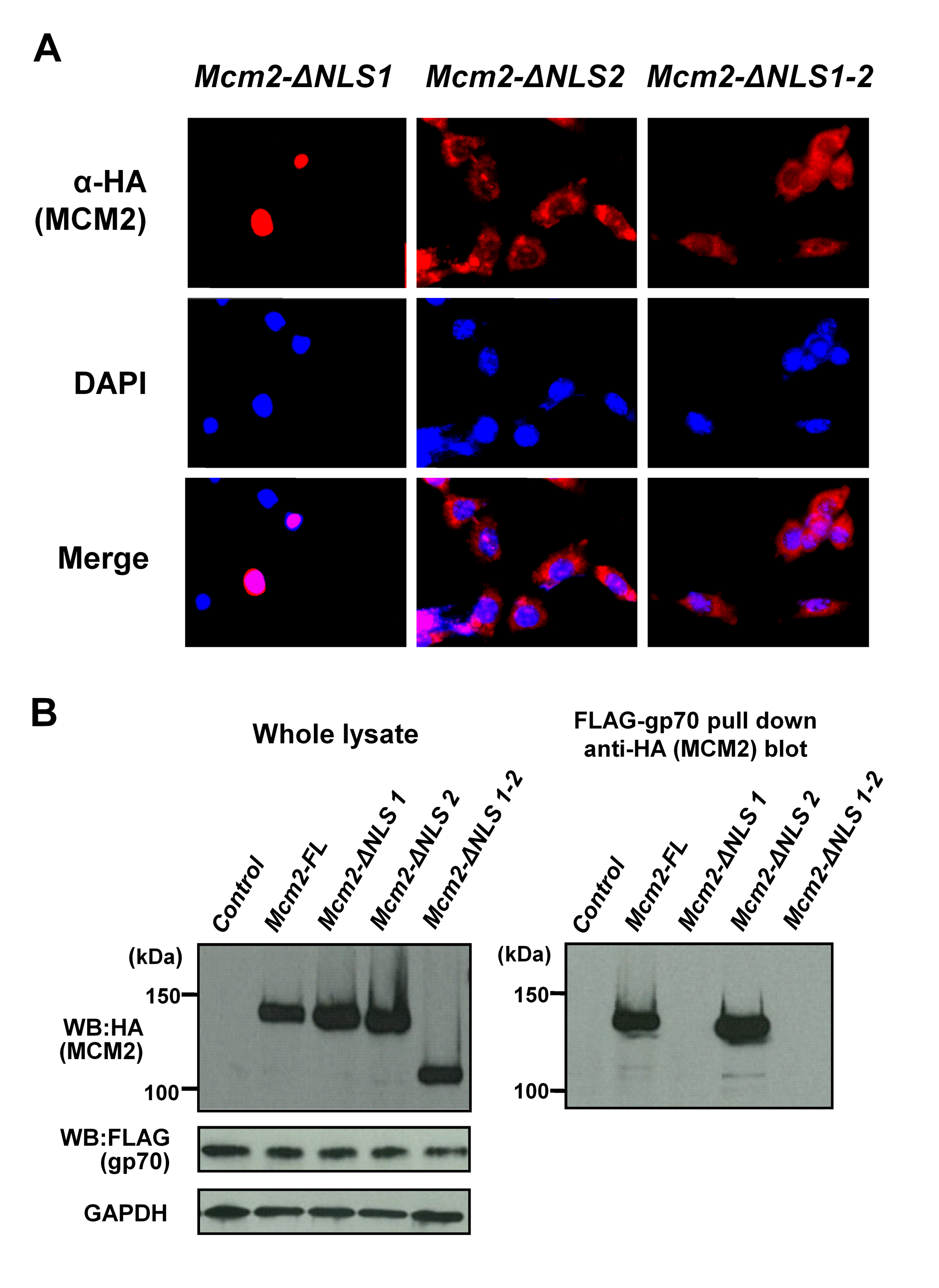

Supplement: Figure S7 — Subcellular localization and interactions of MCM2 NLS deletion mutants in 3T3 cells. (A) 3T3 cells transfected with HA-tagged Mcm2 NLS deletion mutants were treated with 1 µM doxorubicin for 24 h. The cells were then fixed with 1% paraformaldehyde in PBS, permeabilized with 0.1% NP-40 in PBS at room temperature, and stained with TRITC-conjugated anti-HA antibody. HA-positive cells are shown in red (TRITC), and DAPI-stained nuclei are shown in blue. Images were acquired using a BZ-9000 microscope (KEYENCE) with a 400× objective. Note the nuclear localization of MCM2-ΔNLS1 in contrast to the cytoplasmic localization of MCM2-ΔNLS2 and MCM2-ΔNLS1-2. (B) 3T3 cells were transfected with HA-tagged Mcm2 NLS deletion mutants along with FLAG-tagged gp70, and treated with 1 µM doxorubicin for 24 h. Expression of the MCM2 NLS deletion mutants (left panel, upper) and FLAG-gp70 (left panel, middle) was confirmed by western blotting. Lysates from these cells were subjected to a pull-down assay to detect the binding of the MCM2 NLS deletion mutants to FLAG-gp70. MCM2-FL and MCM2-ΔNLS2 proteins coprecipitate with gp70 (right panel). Thus, gp70 is able to interact with MCM2-FL and MCM2-ΔNLS2, but not with MCM2-ΔNLS1 or MCM2-ΔNLS1-2. These results suggest that gp70 is bound to the NLS1 domain of MCM2 and indirectly inhibits the function of NLS2. (TIF) [file pone.0040129.s007.tif]
