# Supplementary material for: Minichromosome Maintenance 2 Bound with Retroviral Gp70 Is Localized to Cytoplasm and Enhances DNA-Damage-Induced Apoptosis
Source: PLoS One. 2012 Jun 29;7(6):e40129. doi: 10.1371/journal.pone.0040129 (PMC3387003; doi:10.1371/journal.pone.0040129)
Supplement: Table S1 — Identification of genes with expression patterns similar to that of Mcm2 using the GeneChip assay. Gene expression patterns were determined by the GeneChip assay in FLV-infected or un-infected C3H/C57BL/6 mice after treatment with doxorubicin. A part of genes exhibited similar expression patterns with Mcm2. The similarity in gene expression patterns was evaluated with the Percellome system using a Pearson product-moment correlation coefficient. (DOCX) [file pone.0040129.s008.docx]

**Table S1. Identification of genes with expression patterns similar to that of *Mcm2* using the GeneChip assay.**

**GeneChip ID Gene symbol GenBank ID**

**(Correlation coefficient >99%)**

1416214_at *Mcm4* BC013094

1415945_at *Mcm5*  NM_008566

1416031_s_at *Mcm7* NM_008568

1424144_at *Cdt1* AF477481

1417019_a_at *Cdc6* NM_011799

1419802_at *Cdca5* NM_026410

1422430_at *Fignl1* NM_021891

1421731_at *Fen1* NM_007999

1418369_at *Prim1* J04620

1415810_at *Uhrf1* BB702754

1417506_at *Gmnn* NM_020567

1416479_a_at *Tmem14c* NM_025387

1416641_at *Lig1* NM_010715

1419270_a_at *Dut* AF091101

1415878_at *Rrm1* BB758819

1449085_at *Phf10* NM_024250

1448627_s_at *Pbk* NM_023209

1454636_at *Cbx5* AV114188

1424321_at *Rfc4* BC003335

1426612_at *Tipin* AK011357

**(Correlation coefficient >90%, extracted)**

1451576_at *Dnapk* D87521

1449839_at *Casp3* BG070529

1417367_at *Ppp2ca* BC003856

1424346_at *Ppp6* BG065679

Genes showing similar expression patterns with *Mcm2* are listed in Table S1. The similarity was evaluated by the percellome system using a Pearson product-moment correlation coefficient of more than 99% (twenty genes). For a correlation coefficient of more than 95%, about 300 genes are listed. For that of more than 90%, about 600 genes are listed. The expression of these genes might be regulated by similar signaling systems with *Mcm2*-associated pathways after DNA-damage and FLV-infection in C3H and C57BL/6 mice.
